# Unveiling adcyap1 as a protective factor linking pain and nerve regeneration through single-cell RNA sequencing of rat dorsal root ganglion neurons

**DOI:** 10.1186/s12915-023-01742-8

**Published:** 2023-10-25

**Authors:** Qi Chen, Xi-Yin Zhang, Yu-Pu Wang, Yun-Jie Fu, Feng Cao, Yi-Nuo Xu, Jin-Ge Kong, Na-Xi Tian, Yu Xu, Yun Wang

**Affiliations:** 1 Neuroscience Research Institute and Department of Neurobiology, School of Basic Medical Sciences, Key Laboratory for Neuroscience, Ministry of Education/National Health Commission, National Health Commission and State Key Laboratory of Natural and Biomimetic Drugs, Peking University, Beijing, 100083 China; 2 PKU-IDG/McGovern Institute for Brain Research, Peking University, Beijing, 100871 China

**Keywords:** Dorsal root ganglion, Neuropathic pain, Axon regeneration, Peripheral nerve injury, Single-cell RNA-seq

## Abstract

**Background:**

Severe peripheral nerve injury (PNI) often leads to significant movement disorders and intractable pain. Therefore, promoting nerve regeneration while avoiding neuropathic pain is crucial for the clinical treatment of PNI patients. However, established animal models for peripheral neuropathy fail to accurately recapitulate the clinical features of PNI. Additionally, researchers usually investigate neuropathic pain and axonal regeneration separately, leaving the intrinsic relationship between the development of neuropathic pain and nerve regeneration after PNI unclear. To explore the underlying connections between pain and regeneration after PNI and provide potential molecular targets, we performed single-cell RNA sequencing and functional verification in an established rat model, allowing simultaneous study of the neuropathic pain and axonal regeneration after PNI.

**Results:**

First, a novel rat model named spared nerve crush (SNC) was created. In this model, two branches of the sciatic nerve were crushed, but the epineurium remained unsevered. This model successfully recapitulated both neuropathic pain and axonal regeneration after PNI, allowing for the study of the intrinsic link between these two crucial biological processes. Dorsal root ganglions (DRGs) from SNC and naïve rats at various time points after SNC were collected for single-cell RNA sequencing (scRNA-seq). After matching all scRNA-seq data to the 7 known DRG types, we discovered that the PEP1 and PEP3 DRG neuron subtypes increased in crushed and uncrushed DRG separately after SNC. Using experimental design scRNA-seq processing (EDSSP), we identified *Adcyap1* as a potential gene contributing to both pain and nerve regeneration. Indeed, repeated intrathecal administration of PACAP38 mitigated pain and facilitated axonal regeneration, while *Adcyap1* siRNA or PACAP6-38, an antagonist of PAC1R (a receptor of PACAP38) led to both mechanical hyperalgesia and delayed DRG axon regeneration in SNC rats. Moreover, these effects can be reversed by repeated intrathecal administration of PACAP38 in the acute phase but not the late phase after PNI, resulting in alleviated pain and promoted axonal regeneration.

**Conclusions:**

Our study reveals that *Adcyap1* is an intrinsic protective factor linking neuropathic pain and axonal regeneration following PNI. This finding provides new potential targets and strategies for early therapeutic intervention of PNI.

**Supplementary Information:**

The online version contains supplementary material available at 10.1186/s12915-023-01742-8.

## Background

Peripheral nerve injury (PNI) frequently occurs in individuals aged 16–59 and poses a widespread problem [1, 2]. In contrast to central nerve injury (CNI), injured neurons and glial cells rapidly initiate the repair process after PNI [3]. However, damaged peripheral nerve fibers possess limited regeneration capacity, particularly as their extension distance increases [4–7]. This limitation results in a series of motor and sensory dysfunctions [8, 9]. Axon disconnection often leads to paresthesia and dysesthesia [10, 11], and injury to the endoneurium or epineurium can aggravate dysesthesia or even intractable pain. Consequently, a primary concern in the clinical treatment of PNI is promoting nerve regeneration while simultaneously alleviating neuropathic pain. However, the intrinsic relationship between nerve regeneration and the development of neuropathic pain after PNI remains unclear, hindering the development of effective treatments for PNI.

Following PNI, injured dorsal root ganglion (DRG) neurons form growth cones [12, 13] and increase peripheral sensitization to continuously transmit nociceptive stimulation to the CNS, contributing to the generation and progression of neuropathic pain [14–16]. This makes DRG a convergence point for pain and regeneration in PNI. Due to the high heterogeneity of neurons, we employed single-cell RNA sequencing (scRNA-seq) and conducted comprehensive investigations into the division of DRG neuron subgroups, providing a necessary foundation for further exploring the underlying connections between pain and regeneration within DRG neurons after PNI [17–19]. However, due to the constraints of existing classic animal models, previous studies have treated neuropathic pain and nerve regeneration as separate entities, which does not align with the practical needs of our research.

In this research, a new rat model called spared nerve crush (SNC) was developed to replicate neuropathic pain and axonal regeneration following PNI. By collecting scRNA-seq data from crushed and uncrushed rat DRG neurons at different time points post-SNC, we found the PEP1 neuronal subtype in the crushed DRG to be particularly notable. Utilizing an experimental design for scRNA sequence processing (EDSSP) and functional validation, we identified a potential key gene, *Adcyap1*, which encodes a crucial molecule providing protection against pain and promoting nerve regeneration during the acute phase after PNI.

Overall, our research suggests that the spared nerve crush rat model can be a valuable resource for investigating the mechanisms of PNI and identifying potential therapeutic targets. Our findings reveal an inherent relationship between neuropathic pain and axonal regeneration following PNI and identify *Adcyap1* as a novel potential molecular target for early therapies aimed at reducing neuropathic pain and promoting nerve regeneration. This provides new insights into the molecular processes involved in PNI.

## Results

### The SNC rat model successfully replicates both neuropathic pain and axonal regeneration following PNI

**Fig. 1:**
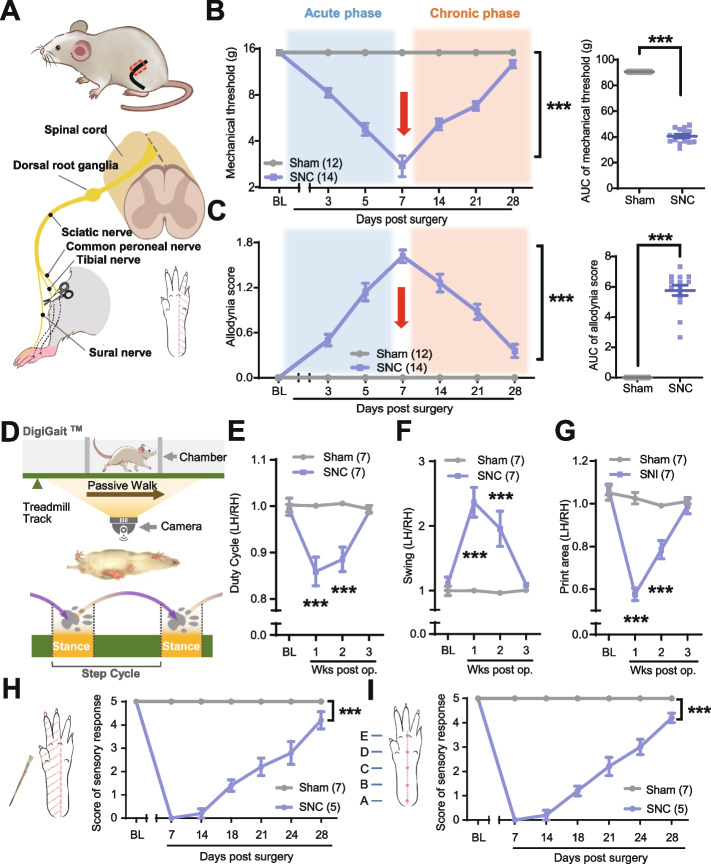
Recovery of sensory and motor functional in the SNC rat model. **A** Diagram illustrating the surgical procedures for creating the SNC rat model (left) and the sites of neuropathic pain testing in the hind paw (bottom right). **B**, **C** Time course of mechanical threshold (**B**) and allodynia score (**C**) for the ipsilateral hind paw in the sham (*n* = 12) and SNC (*n* = 14) groups before and after nerve injury; the area under the curve (AUC) analysis (3–28 days) is displayed in the right panel. **D** Schematic of the DigiGait™ system and method for measuring the duty cycle during gait analysis. **E**–**G** Time course of the injured left hind/right hind (LH/RH) ratio of the duty cycle (**E**), swing (**F**), and print area (**G**) for the sham (*n* = 7) and SNC (*n* = 7) groups before and after nerve injury. **H** A schematic diagram illustrating the site of gentle brush stimulation on the hind paw (left) and the time course of light touch sensitivity of the medial side of the ipsilateral hind paw for the sham (*n* = 7) and SNC (*n* = 5) groups before and after nerve injury (right). **I** A schematic diagram demonstrating the site of pinprick stimulation on the hind paw (left) and the time course of pinprick sensitivity on the medial side of the ipsilateral hind paw for the sham (*n* = 7) and SNC (*n* = 5) groups before and after nerve injury (right). SNC, spared nerve crush. Data are represented as mean ± SEM. Two-way ANOVA with Sidak’s multiple-comparisons test was used in the left panels of **B** and **C**, **E**, **F**, **G**, **H**, and **I**. ***P < 0.001; unpaired *t* test was used in the right panels of **B** and **C**. ***P < 0.001

To study neuropathic pain and axonal regeneration following peripheral nerve injury (PNI), we used a rat model that combines the spared nerve injury (SNI) model (Fig. 1A), a classic neuropathic pain rodent model, with the sciatic nerve lesion model, a typical nerve injury regeneration rodent model [20, 21]. The SNC model causes less damage than other nerve repair models, facilitating the observation of the tibial nerve (TN) and common peroneal nerve (CPN) nerve repair process. Moreover, the SNC model retained an uninjured branch of the sciatic nerve, providing a structural basis for studying neuropathic pain after PNI. As a result, the dorsal root ganglion (DRG) neurons in the SNC model can be categorized into two groups: the crushed neurons of the TN and the CPN, involved in nerve regeneration, and the uncrushed neurons of the sural nerve (SN), which mediated pain transmission.

To validate the SNC model, we employed various methods to examine the animals’ functional recovery after injury. Initially, the *von Frey* test or brush test was used to assess punctate allodynia or A fiber-related tactile allodynia [22]. In SNC rats, both types of allodynia were progressively worsened within 7 days post-injury (dpi), making 7 dpi as a turning point for SNC animal pain behavior (Fig. 1B, C). Consequently, we categorized the post-injury period into two phases: the acute phase (prior to 7 dpi) and the chronic phase (post 7 dpi). The motor function of rats after SNC was also assessed. The pawprint area, duty cycle, and swing of the injured hind paw displayed significant abnormalities in the SNC group before 2 weeks post-injury (wpi) (*P* < 0.05 vs. the sham group; Fig. 1E–G). However, these parameters normalized at 3 wpi (*P* > 0.05 vs. the sham group; Fig. 1E–G), indicating the recovery of weight-bearing ability (Fig. 1D).

Sensory impairment caused by denervation was evaluated using pinprick and light touch sensitivity tests on the injured TN and CPN innervating the plantar area. The findings revealed that both types of touch sense gradually recovered in SNC rats within 28 dpi (Fig. 1H, I). Reinnervation of the TN and the CPN was confirmed by immunofluorescence staining of plantar skin biopsies, indicating the axons regenerated in SNC rats in vivo. No significant difference was observed between sham and SNC rats at 28 dpi (*P* > 0.05 vs. the sham group; Additional file 1: Fig. S1). Therefore, the SNC model successfully replicates neuropathic pain and axonal regeneration after PNI, allowing the study of the intrinsic relationship between these two crucial biological processes.

### scRNA-seq preprocessing reveals innate cell heterogeneity among DRG neurons

**Fig. 2:**
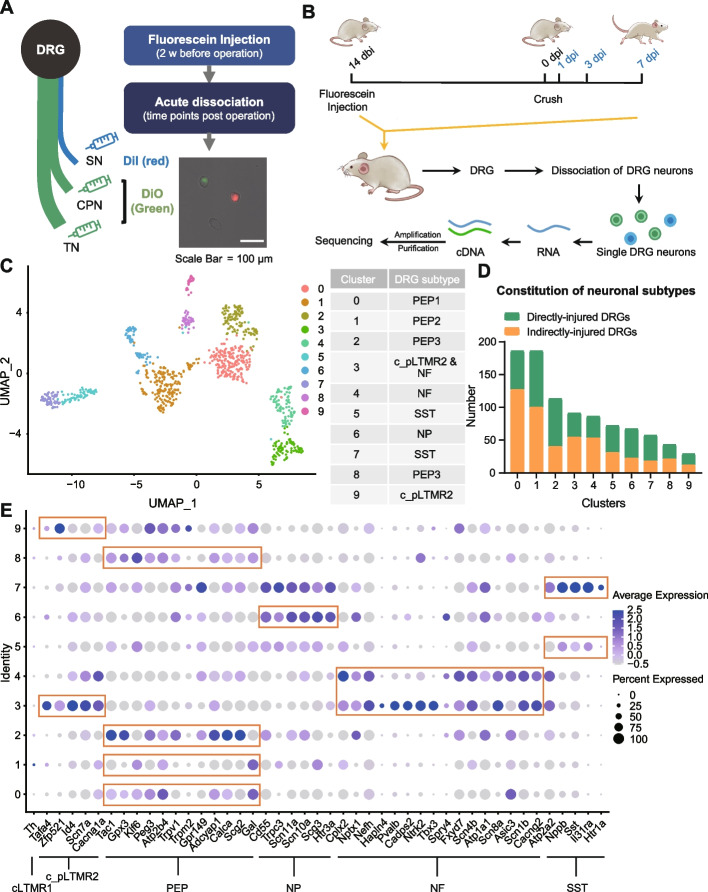
Experimental design and scRNA sequencing preprocessing (EDSSP) revealed that DRG neurons have innate subtypes. **A** Schematic diagram of the fluorescein labeling process and representative image of acute-dissociated DRG neurons labeled with DiO (green) and DiI (red). Scale bar, 100 μm. **B** Schematic diagram of the procedure used to rapidly dissociate and isolate single-labeled DRG neurons for single-cell RNA-seq profiling. dbi, days before injury; dpi, days post-injury. **C**, **D** Unsupervised cell clustering is processed by Seurat and visualized by UMAP. The 10 clusters were annotated based on known/verified cluster markers and sorted into 5 known innate subtypes. **E** Gene expression dot map of the 10 clusters. DRG, dorsal root ganglion; DRGs, dorsal root ganglion neurons; PEP, peptidergic nociceptor; NF, *Nefh*^+^ A-fiber low-threshold mechanoreceptor; NP, nonpeptidergic nociceptor; SST, somatostatin^+^ pruriceptors; c_pLTMR, putative low-threshold c-mechanoreceptor; cLTMR, C-fiber low-threshold mechanoreceptors

To investigate neuropathic pain and axonal regeneration and following PNI, single-cell RNA sequencing was applied to DRG neurons of the SNC rat model. Firstly, different fluorescent dyes were injected into the branches of the rat sciatic nerve before the crush operation. the TN and CPN, representing crushed DRG neurons, were labeled with DiO (green), while the SN, representing the uncrushed group, was labeled with DiI (red) (Fig. 2A). The L4 and L5 DRG neurons on the operated side were acutely separated after recovery, and individual DRG neurons were obtained using a mouth pipette. Neurons of various diameters were collected at 1 day, 3 days, and 7 days after SNC (Fig. 2B). Approximately equal numbers of green and red fluorescent-labeled cells were obtained from each animal. Using a protocol developed by Tang’s lab [23, 24], we performed scRNA-seq on 936 fluorescently labeled DRG neurons and 512 DRG neurons for subtype annotation and time point-related proportions. Of these,1430 cells passed strict quality control (QC) and were used in the subsequent analysis (Additional file 2: Fig. S2).

To obtain the unsupervised cell clustering results, fluorescent-labeled DRG neurons were clustered using a graph-based cluster method. Dimensionality reduction using uniform manifold approximation and projection (UMAP) revealed 10 cell population clusters (Fig. 2C, D). Based on the expression patterns of specific marker genes, these 10 cell clusters were divided into 5 known DRG types [18]: peptidergic nociceptors (PEPs), nonpeptidergic nociceptors (NPs), *Nefh*^+^ A-fiber low threshold mechanoreceptors (NFs), somatostatin^+^ pruriceptors (SSTs), and putative low-threshold c-mechanoreceptors (c_pLTMR2). The expression levels of marker genes in each DRG type are presented in a dot plot (Fig. 2E). PEP DRG neurons were further categorized into PEP1 (*Tac1*- and *Gal*-expressing), PEP2 (*Gal*- and low *Tac1*-expressing), and PEP3 (*Tac1*- and *Gpx3*-expressing with very low levels of *Gal* expression). Consequently, we aligned all single-cell data with 7 known DRG subtypes, which were ultimately consolidated into 6 DRG neuron groups due to marker overlap caused by a limited cell number.

### PEP1 is significantly associated with injury and repair processes

**Fig. 3:**
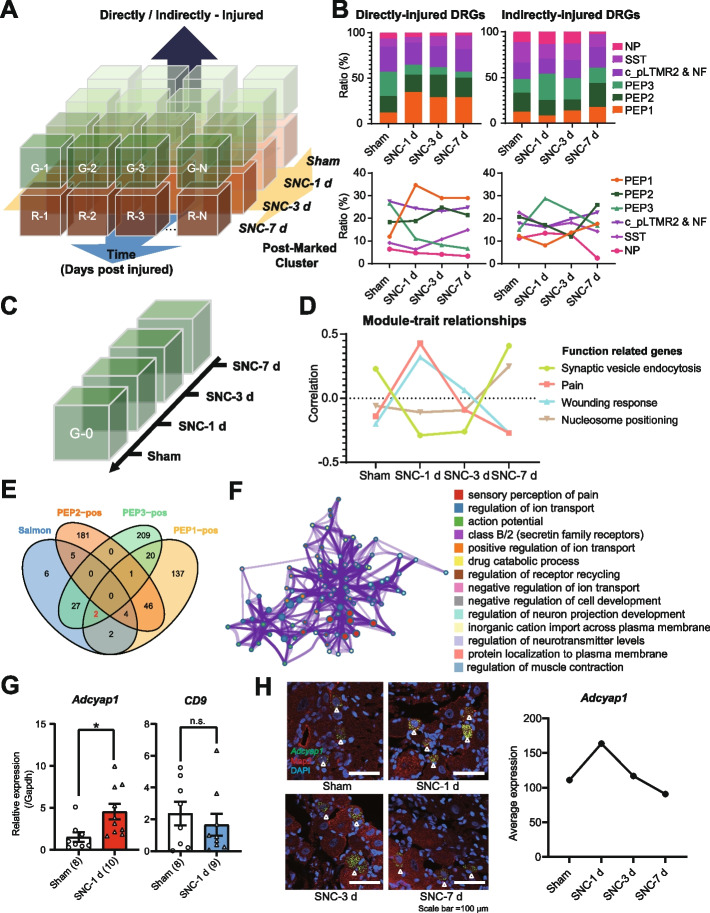
EDSSP indicated that Adcyap1 is likely highly associated with injury and repair processing. **A** The model of the first step of EDSSP. Processed by Seurat, the DRG neurons are sorted by known/verified cluster markers into several clusters and then sorted by time-design and surgical-design into several sub-modules. **B** The ratio changes of sub-types DRG neurons (DRGs) show that PEP1 may relate with injury and repair processing. **C** The model of the second step of EDSSP. Processed by WGCNA, the crushed DRG neurons of PEP1 are sorted into 4 time points according to the experimental design. **D** Module-trait relationships reveal that there are 4 modules of genes changed by time-design. **E** Venngram of Pain genes and 3 PEP subtypes’ high-expression genes. **F** The metascape results of Pain module genes. **G** The expression of *Adcyap1* (left) and *CD9* (right) in the PEP1 DRGs for the sham (*n* = 8) and SNC-1 d (*n* = 10 for *Adcyap1*; *n* = 9 for *CD9*) groups by single-cell qPCR, unpaired *t* test. *P < 0.05. n.s., no significant difference. **H** RNAscope® images of rat DRGs stained with probes and antibody (left), *Adcyap1* (green), Map2 (red), and DAPI (blue). Representative sections from sham and SNC 1-day, 3-day, and 7-day DRGs are shown. RNAscope statistical graph of *Adcyap1* in different time points after SNC (Right)

By suitably decreasing the clustering resolution, we could recognize subtypes of DRG neurons while maintaining the intragroup disparities in the cell subsets, laying the groundwork for further analysis. To explore the mechanism responsible for axonal regeneration and neuropathic pain development after PNI, we assessed DRG neurons in three dimensions: the intrinsic subtype of DRG neurons to which they belonged, whether the DRG neuron was injured, and the time post-injury based on the experimental design (Fig. 3A).

In the damaged DRG, we discovered that a group of DRG neurons corresponding to PEP1 accounted for a larger proportion of cells 1 day after SNC, while the share of neurons matching to PEP2 slightly increased 3 days post-SNC. Among the uninjured DRG, the proportion of PEP3 DRG neurons increased 1 day after SNC (Fig. 3B). Adult DRG neurons are mature cells that cannot divide. The increase in the proportions of certain DRG neuron subtypes following SNC indicates that some neurons that originally belonged to other DRG subtypes might have switched to these subtypes. Thus, we hypothesize that the PEP1 subset of damaged DRG neurons might play a significant role in axonal regeneration after PNI, while PEP3 neurons in uninjured DRG could be closely associated with pain transmission during neuronal regeneration.

To verify this hypothesis, we employed Metascape [25] to conduct Gene Ontology (GO) and Kyoto Encyclopedia of Genes and Genomes (KEGG) analyses on DRG neurons belonging to the three PEP subsets. A substantial number of genes expressed in PEP1 DRG neurons were concentrated in categories related to axonal regeneration, including response to wounding, positive regulation of cell projection organization, and positive regulation of cellular component biogenesis (Additional file 3: Fig. S3 and Additional file 4: Fig. S4A). The genes expressed in PEP3 DRG neurons were prominently present in categories associated with neuropathic pain, such as sensory perception of pain, regulation of membrane potential, and inorganic cation transmembrane transport (Additional file 4: Fig. S4B). We also examined the functions of genes expressed in PEP2 DRG neurons, but discovered that many were linked to nonspecific post-injury biological events (Additional file 4: Fig. S4C). These results indicate that the PEP1 subset of neurons in crushed DRG plays a crucial role in axonal regeneration following PNI, while PEP3 neurons in uncrushed DRG are involved in pain transmission during nerve regeneration.

### Experimental design scRNA-seq processing (EDSSP) reveals 4 gene modules that are highly related to injury and pain processing

Based on the SNC model, which links neuropathic pain and axonal regeneration, we obtained a large amount of high-quality transcript information for individual DRG neurons in response to physiological conditions. To address this, we established an experimental design scRNA-seq processing (EDSSP) and used it for corresponding bioinformatics analysis of our high-quality sample data.

In 2016, researchers employed WGCNA to investigate the functions of DRG neurons and explored gene co-expression modules within DRG neuronal subtypes. This study implied that multiple tools could be combined to analyze transcriptome data [19]. Consequently, a combination of gene-based clustering patterns and cell-salient characterization-based clustering patterns was implemented using WGCNA [26, 27] and Seurat [28]. Moreover, gene co-expression clustering was conducted using the timeline of in vivo recovery of crushed PEP1 DRG neurons as a characterization group. These approaches collectively constituted the complete intrinsic logic of the EDSSP (Fig. 3C and Additional file 5: Fig. S5A-B). Through the aforementioned procedures, 14 gene modules were obtained, including 4 modules closely associated with temporal information and gene function. The modules were named according to the highest hit gene number’s items in GO and KEGG (Fig. 3D–F, and Additional file 5: Fig. S5C-E). Among them, the Pain and Wounding response modules were significantly upregulated at 1 dpi, while the genes in the Greenyellow and Tan modules were significantly upregulated at 7 dpi (Fig. 3D).

Furthermore, we found that a large number of genes in the Pain module were enriched in the categories related to PNI, such as pain sensory perception, ion transport regulation, and regulation of neuron projection development (Fig. 3F). Among them, *trpv1* and *trpa1*, genes encoding two classic TRP channels involved in the regulation of pain and itching, and *Scn10a*, encoding the voltage-gated sodium channel Na_v_1.8 and associated with the upstroke of the action potential, have been well studied [29–32]. Also, we discovered that at 7 dpi, the turning point of SNC animal pain behavior, abundant gene transcription, and protein synthesis were occurring in DRG neurons at this time (Additional file 5: Fig. S5D-E). These findings also validate that EDSSP can facilitate the integration of actual post-injury time dimension or other design parameters into co-expression analysis, thereby motivating us to explore pertinent alterations in gene expression through this combination of techniques.

### Adcyap1 provides protection against pain and promotes nerve regeneration during the acute phase following PNI

Through EDSSP, we identified the Pain module, a group of genes with expression significantly correlated with post-injury time points. Additionally, PEP1 DRG neurons are significantly associated with axonal regeneration, while PEP3 neurons are related to neuropathic pain. Therefore, to explore the potential target genes linking neuropathic pain to axonal regeneration following PNI, we analyzed the intersection of differentially expressed genes of the PEP1 and PEP3 DRG neurons using the Pain module.

As the target gene scope was narrowed down, two potential key mediator genes, *Adcyap1* and *CD9*, were identified. These genes showed increased expression at 1 dpi (Fig. 3F). This prediction was confirmed through single-cell qPCR, revealing that *Adcyap1* expression was significantly upregulated in PEP1 DRG neurons at 1 dpi (*P* < 0.05 vs. the sham group; Fig. 3G left). However, there was no significant difference in *CD9* expression at that time (*P* > 0.05 vs. the sham group; Fig. 3G right). This difference might be attributed to substantial variations in *CD9* expression in naïve PEP1 DRG neurons. Subsequently, we found that *Adcyap1* expression was highest in SNC-1 d and decreased until the 7 days after SNC by RNAscope (Fig. 3H).

*Adcyap1* is a conserved gene in humans, rats, and mice [33]. It is located on chromosome 18 and encodes a secreted neuropeptide called pituitary adenylate cyclase-activating polypeptide (PACAP), which participates in various biological functions, including controlling anterior pituitary hormone secretion, insulin secretion, vasodilation, and immunosuppression [34, 35]. Three PACAP receptors, VPAC1, VPAC2, and PAC1, have been identified; all of them are G protein-coupled receptors located on the cell membrane [36]. Our bioinformatics analysis suggests that *Adcyap1* is likely involved in both neuropathic pain and nerve regeneration after PNI.

**Fig. 4:**
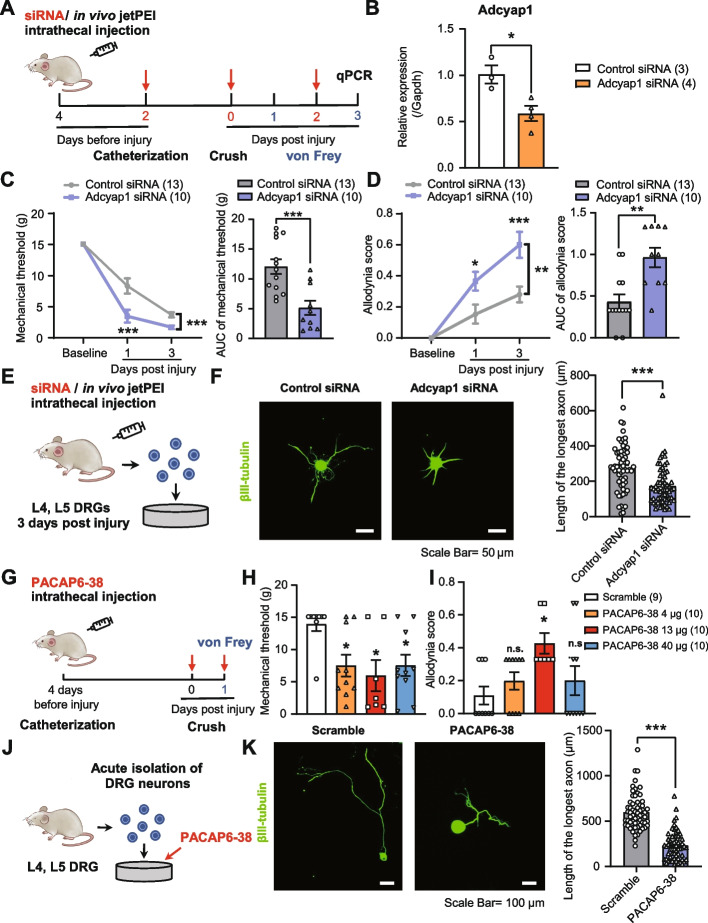
Adcyap1 siRNA or PACAP6-38 promotes pain hypersensitivity and inhibits axonal outgrowth following SNC. **A** Schematic diagram for the measurement of nociceptive responses after intrathecal administration of *Adcyap1* siRNA. **B** The expression of *Adcyap1* in DRGs of the control siRNA (*n* = 3) and *Adcyap1* siRNA (*n* = 4) groups. **C**, **D** Time course of mechanical threshold (**C**) and allodynia score (**D**) of the ipsilateral hind paw of the control siRNA (*n* = 13) and *Adcyap1* siRNA (*n* = 10) groups, with area under the curve (AUC) analysis (1–3 days) in the right panel. **E** Schematic diagram for the investigation of axonal outgrowth of DRG neurons after intrathecal administration of *Adcyap1* siRNA. **F** Representative images and quantification of axonal outgrowth in acute-dissociated DRG neurons at 3 days post-SNC of the control siRNA (*n* = 3) and *Adcyap1* siRNA (*n* = 3) groups. Scale bar, 50 μm. **G** Schematic diagram for the measurement of nociceptive responses after intrathecal administration of Pacap6-38. **H**, **I** Mechanical threshold (**H**) and allodynia score (**I**) of the ipsilateral hind paw at 1 day post-SNC for the scramble (*n* = 9), 4 μg PACAP6-38 (*n* = 10), 13 μg PACAP6-38 (*n* = 7), and 40 μg PACAP6-38 (*n* = 10) groups. **J** Schematic diagram for the investigation of axonal outgrowth of DRG neurons clustered with Pacap6-38. **K** Representative images and quantification of axonal outgrowth in acute-dissociated DRG neurons from naïve rats cultured with scramble (*n* = 3) or PACAP6-38 (*n* = 3) for 3 days. Scale bar, 100 μm. Data are represented as mean ± SEM. Unpaired *t* test in **B**, the right panel of **C** and **D**, **F**, and **K**; two-way ANOVA with Sidak’s multiple-comparisons test in the left panel of **C** and **D**; one-way ANOVA with Tukey’s multiple comparisons test in **H** and **I**. *P < 0.05, **P < 0.01, ***P < 0.001. n.s., no significant difference

First, we examined the function of *Adcyap1* and PACAP by intrathecally injecting *Adcyap1* siRNA into SNC rats and assessing their punctate and dynamic allodynia in response to mechanical stimuli (Fig. 4A). The results showed that intrathecal injection of *Adcyap1* siRNA decreased the mRNA levels of *Adcyap1* in DRG neurons (*P* < 0.05 vs. the control siRNA group; Fig. 4B) and silencing of Adcyap1 in DRG neurons with siRNA worsened punctate and dynamic allodynia within 3 days after SNC (*P* < 0.001 or *P* < 0.01 vs. the control siRNA group; Fig. 4C, D). Additionally, we extracted the L4 and L5 DRG from the rats 3 dpi post-injection, dissociated DRG neurons, and cultured them for 2 days (Fig. 4E). We observed that axonal outgrowth from the DRG neurons was significantly inhibited after intrathecal injection of *Adcyap1* siRNA (*P* < 0.001 vs. the control siRNA group; Fig. 4F). Moreover, we administered PACAP6-38, an antagonist of PAC1R, into SNC rats (Fig. 4G); we observed a bell-shaped, dose-dependent increase in the punctate and dynamic allodynia upon repeated intrathecal injection of PACAP6-38 during the acute phase of PNI (Fig. 4H, I). Finally, we investigated the necessity of PACAP for axonal regrowth by adding PACAP6-38 (antagonist of PAC1R) or PACAP38 (agonist of PAC1R) to the medium used for culturing acutely dissociated DRG neurons from adult naïve rats (Fig. 4J). Our results indicated that PACAP6-38 inhibited axonal outgrowth (*P* < 0.001 vs. the scramble peptide group; Fig. 4K), while PACAP38 promoted axonal outgrowth of primary cultured DRG neurons (*P* < 0.01 vs. the scramble peptide group; Additional file 6: Fig. S6A-B).

**Fig. 5:**
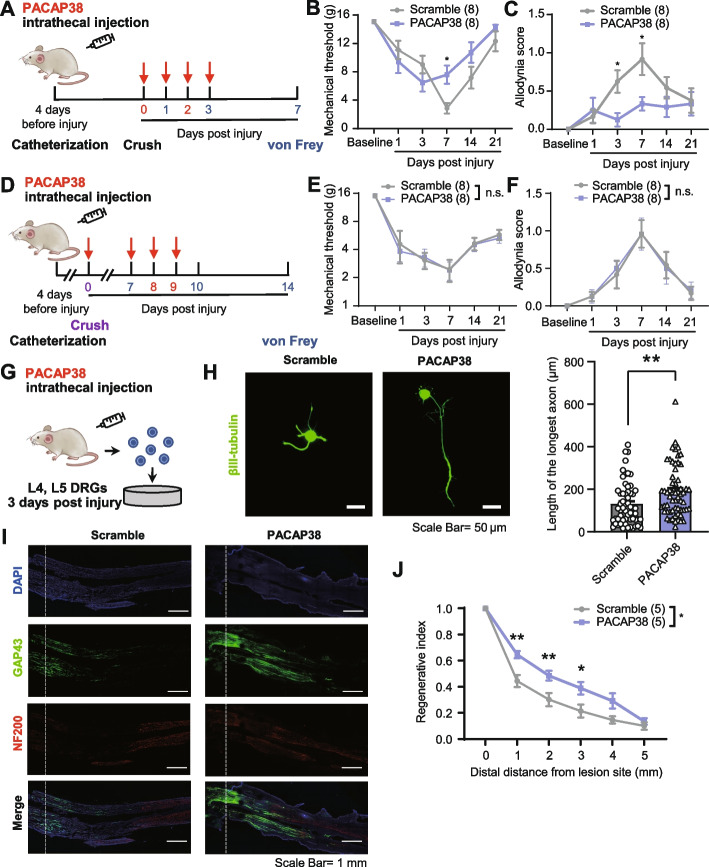
Repeated intrathecal injection of PACAP38 attenuates mechanical allodynia and promotes axonal regeneration following SNC. **A** Schematic diagram for the measurement of nociceptive responses after intrathecal administration of PACAP38 in the acute phase of SNC. **B**, **C** Time course of mechanical threshold (**B**) and allodynia score (**C**) of the ipsilateral hind paw after intrathecal administration of scramble (*n* = 8) or PACAP38 (*n* = 8) in the acute phase of SNC. **D** Schematic diagram for the measurement of nociceptive responses after intrathecal administration of PACAP38 in the chronic phase of SNC. **E**, **F** Time course of mechanical threshold (**E**) and allodynia score (**F**) of the ipsilateral hind paw after intrathecal administration of scramble (*n* = 8) or PACAP38 (*n* = 8) in the chronic phase of SNC. **G** Schematic diagram for the investigation of axonal outgrowth of DRG neurons after intrathecal administration of PACAP38. **H** Representative images and quantification of axonal outgrowth in acute-dissociated DRG neurons from rats at 3 days post-SNC for the scramble (*n* = 3) and PACAP38 (*n* = 3) groups, immunostained for βIII-tubulin. Scale bar, 50 μm. **I** Representative images of longitudinal sections of the two crushed sciatic nerve branches (TN and CPN) in the scramble and PACAP38 groups at 3 dpi, immunostained for GAP43 (regenerated nerve fibers) and NF200 with DAPI staining. Scale bar, 1 mm. The dashed line indicates the crush site. **J** Quantification of axonal regeneration with the regeneration index measured as GAP43 intensity normalized to the crush site for the scramble (*n* = 5) and PACAP38 (*n* = 5) groups. DGRs, DRG neurons. Data are represented as mean ± SEM. Two-way ANOVA with Sidak’s multiple-comparisons test in **B**, **C**, **E**, **F**, and **J**; unpaired t-test in **H**. *P < 0.05, **P < 0.01, ***P < 0.001. n.s., no significant difference

**Fig. 6:**
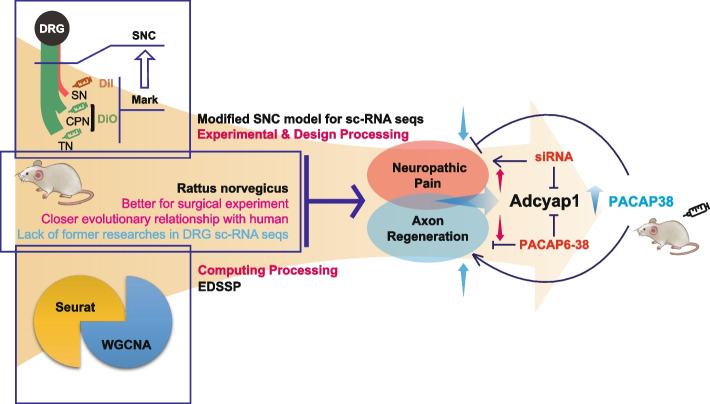
Working model. By performing scRNA-seq on crushed and uncrushed DRG neurons from SNC rats, an animal model established as a basis for studying the relationship between evoked neuropathic pain and nerve regeneration after peripheral nerve injury, DRG neurons were matched to 7 known subtypes; among these, the PEP1 neuronal subtype in crushed DRG is particularly interesting. After EDSSP and functional verification, the key gene Adcyap1 was found to have a protective effect on pain and nerve regeneration. This study provides new insights into the intrinsic link between neuropathic pain and axonal regeneration following PNI and offers new molecular targets and ideas for early clinical treatment

To further confirm the protective effect of PACAP38 in vivo, we administrated PACAP38 intraoperatively and continued treatment until 3 dpi, the acute phase. We found that the punctate and dynamic allodynia of the SNC rat was attenuated at 7 dpi and lasted until 21 dpi (*P* < 0.05 vs. the scramble peptide group; Fig. 5A–C, and P < 0.05 vs. the scramble peptide group; Additional file 6: Fig. S6C). However, when we administrated PACAP38 intraoperatively with a 3-day lasting administration from 7 to 9 dpi, which is in the chronic phase, the effect of PACAP38 on mechanical allodynia disappeared (*P* > 0.05 vs. the scramble peptide group; Fig. 5D–F). Additionally, intrathecal injection of Pacap38 in the acute phase also promoted axonal outgrowth of DRG neurons (*P* < 0.01 vs. the scramble peptide group; Fig. 5G, H) and axonal regeneration of the SNC rat at 3 days after injury (*P* < 0.05 vs. the scramble peptide group; F ig. 5I, J). These findings indicate that *Adcyap1* provides protection against neuropathic pain and accelerates axonal regeneration during the acute phase of PNI, but not in the chronic phase. This discovery uncovers an inherent connection between neuropathic pain and axonal regeneration following PNI, opening up new possibilities for early therapeutic intervention (Fig. 6).

## Discussion

Patients suffering from PNI experience motor and sensory dysfunctions that severely affects their quality of life [37]. When the window for peripheral nerve regeneration has closed, active nerve regeneration can lead to the persistence of neuropathic pain after PNI [38, 39], emphasizing the need for more effective early therapeutic interventions. However, due to the limited understanding of the mechanisms linking axonal regeneration and neuropathic pain after PNI, it remains unclear how to promote nerve regeneration while minimizing the risk of neuropathic pain during clinical treatment. Although nerve coaptation has been shown to prevent neuropathic pain by promoting nerve regeneration, it requires advanced surgical skills and is thus not widely applicable [40, 41]. Additionally, in PNI studies, researchers typically examine axonal regeneration and neuropathic pain separately, and established animal models may not accurately represent PNI patients’ conditions. To address this issue, we initially developed a selective nerve crush (SNC) rat model where the epineurium remained intact and the two branches of the sciatic nerve were selectively crushed, thereby successfully replicating axonal regeneration and neuropathic pain to PNI patients’ experiences. The SNC model enables the differentiation between DRG neurons associated with axonal regeneration and those connected to neuropathic pain, aiding subsequent research. This research serves as a crucial complement to single-cell sequencing data obtained from DRG research on rats and previous studies in mice [17–19]. By leveraging the SNC design, we effectively utilized retrograde fluorescent labeling to categorize DRG neurons that project axons into the sciatic nerve into two groups: the crushed group, primarily responsible for axonal regeneration, and the uncrushed group, largely associated with neuropathic pain. This classification significantly facilitated our subsequent inquiries.

Over the past 20 years, the significant advancements in sequencing technologies have allowed single-cell transcriptomics to address questions that are distinct from those typically addressed by bulk transcriptomics. This approach is particularly suitable for neuroscience research involving nerve systems with significant cellular heterogeneity [42–45]. Concurrently, research on DRG neuron subtypes has made significant progress, laying an essential foundation for further exploration of the specific functions of DRG neurons [17, 18]. Following SNC, DRG neurons, at the crossroads of pain and regeneration, present an attractive model for examining the relationship between pain and nerve regeneration in PNI. In our research, we obtained scRNA-seq data from single neurons isolated from SNC rats at various post-injury time points. The DRG neurons we collected all projected their axons into the sciatic nerve and were either directly or indirectly influenced by the crush injury. Additionally, we established the connection between neuropathic pain and axonal regeneration prior to sequencing. The utilization of single-cell capture via mouth pipette provided optimal conditions for determining the transcript features of DRG neurons. By mapping all single-cell data to 7 known DRG subtypes, we inferred that the PEP1 subset of crushed DRG neurons may play an essential role in axonal regeneration, while PEP3 neurons in uncrushed DRG neurons were expected to be closely associated with pain transmission, based on changes in the proportions of different subtypes of neurons that occurred after SNC.

We sorted the 10 observed rat DRG neuron clusters into 7 established DRG neuronal subtypes: PEP1 (*Tac1*^+^/*Gal*^+^), PEP2 (*Gal*^+^ with low *Tac1* expression), PEP3 (*Tac1*^+^/*Gpx3*^+^ with low *Gal* expression), NP (*Scg3*^+^ with low *Sst* expression), NF (*Nefh*^+^), SST (*Sst*^+^), and c_pLTMR2 (*Zfp521*^+^ with very low *Th* expression). Due to limited cell numbers and marker overlap, the 7 established DRG neuronal subtypes were ultimately consolidated into 6 DRG neuron groups. It should be noted that the DRG subpopulation division in rats is slightly different from that reported in mice and has been confirmed and annotated through follow-up DRG scRNA sequencing in rats. Galanin (*Gal*), a modulator of neurotransmission in the central and peripheral nervous systems [46], associated with feelings, pain, and regulation of anterior pituitary hormone production, energy and osmotic homeostasis, reproduction, and cognition [47, 48] and serve as a marker for PEP1 neurons. Secretogranin III (*Scg3*), a marker for secretory granules (SGs) involved in the transport and metabolism of the parkinsonian toxin PQ, which can lead to the injury of multiple intracellular organelles in dopamine neurons [49], is a marker for NP neurons. Additionally, *Zfp521*, a Runx1-dependent transcription factor implicated in neural development [50], is considered one of the marker genes for c_pLTMR2 neurons. In line with this discovery, *Zfp521* was previously found to be expressed in a type of Runx1-dependent neuron, *VGLUT3*^+^ cLTMR [51]. Therefore, our research offers valuable insights into the gene expression map of rat DRG intrinsic subtype classification. However, caution should be exercised when employing this matching method if significant evolutionary exists between germlines.

In contrast to the automatic sorting of single cells by machines, the limited quantity of high-quality samples in our research presented a challenge for bioinformatics analysis. Previous studies have employed weighted gene co-expression network analysis (WGCNA) to examine the correlation between cell transcripts in DRG subtypes involved in diverse biological processes [26]. This prompted us to integrate WGCNA with Seurat to explore the gene expression differences in DRG neuronal subtypes, from both intrinsic subtypes and experimental design viewpoints. To overcome the constraints of single-cell transcriptomics experiments, which may not always align with biological and medical realities, we suggest a combinatorial bioinformatics analysis approach, EDSSP. However, the applicability of EDSSP may be limited without basic research on DRG neuron classification, which could affect the stability and reliability of subsequent analyses. Therefore, we suggest that researchers verify the presence of a reasonable and widely accepted subtype gene expression profile of the corresponding tissue or cell before employing EDSSP. Overall, EDSSP provides a solution for addressing complex scientific problems. Modified bioinformatics analysis of single-cell transcriptomes holds great potential for future neuroscience research.

By analyzing the EDSSP of differentially expressed genes in crushed PEP1 neurons, we identified 4 gene modules with significant differences in their expression time courses. Among them, the genes within the Pain module are particularly noteworthy, as they contain two potential mediator genes, *Adcyap1* and *CD9*, that may be involved in both pain and nerve regeneration. Single-cell qPCR confirmed *Adcyap1* as a promising candidate for further study. Subsequent experiments showed that intrathecal injection of *Adcyap1* siRNA or PACAP6-38, an antagonist of PAC1R, inhibited axonal outgrowth. In contrast, administration of PACAP38, a transcriptional product of *Adcyap1*, promoted axonal outgrowth both in vitro and in vivo. Our findings are consistent with previous studies indicating upregulated transcription and translation of *Adcyap1* in rat DRG after sciatic nerve injury and the promotion of axon outgrowth in human sensory neurons by PACAP38 [52–55]. However, we observed that intrathecal injection of *Adcyap1* siRNA or PACAP6-38 induced severe mechanical hyperalgesia in SNC rats, suggesting that *Adcyap1* might play different roles in the central and peripheral nervous systems. Future studies could explore the local administration of Adcyap1 in the peripheral regions to avoid adverse reactions in other systems. Notably, intrathecal injection of PACAP38 during the acute phase of SNC attenuated mechanical allodynia and promoted axonal regeneration, indicating that Adcyap1 has significant implications for intraoperative intervention in PNI patients. Interestingly, PACAP38 has been identified as a promising therapeutic agent for diabetic neuropathy [56]. Overall, our findings suggest that *Adcyap1* could potentially serve as an intrinsic protective factor promoting nerve regeneration and preventing pain during the acute phase after PNI.

## Conclusions

Our research uncovers an inherent protective factor, *Adcyap1*, connecting neuropathic pain and axonal regeneration following PNI. This discovery offers new potential targets and strategies for early therapeutic intervention in cases of PNI.

## Methods

### Animals

Specific pathogen-free (SPF) male Sprague–Dawley (SD) rats weighing 200 g were provided by the Animal Center of Peking University Health Science Center. The animals were housed in an SPF laboratory in ventilated cages (IVC) (3–5 rats per cage) on a 12-h light–dark cycle (lights on at 8:00 AM) with free access to food and water. All experiments were performed in accordance with the guidelines of the Animal Care and Use Committee of Peking University, with efforts made to minimize the discomfort of the animals.

### Spared nerve crush (SNC)

During the operation, the rats were anesthetized using isoflurane, and a 2-cm incision was made on the skin at the mid-thigh level of the left thigh to expose the left sciatic nerve. The three branches of the nerve were carefully separated using a glass dissecting needle, and the tibial and common peroneal nerves were crushed for 30 s using 3 clicks of an ultrafine microscope forceps (WA2030, JinZhong, Shanghai, China), leaving the remaining sural nerve intact. The muscle and skin were then closed in two layers, and penicillin was applied to the wound. For the sham operation, the sciatic nerve was exposed but left uninjured. Animals with marked weight loss or severe immobility at 7 days post-injury were excluded from the study. For the medial plantar sensory recovery (pinprick and light touch sensitivity) test and skin biopsy, we exposed the saphenous nerve above the knee region and ligated and transected it after SNC, making the medial plantar epidermis innervated only by regenerated sciatic nerve axons (Fig. 1A).

### Punctate allodynia (von Frey) test

All behavioral experiments were conducted in a double-blind manner. We conducted the punctate allodynia test to evaluate the paw withdrawal threshold in response to a mechanical stimulus produced by the application of a series of von Frey fibers (Aesthesio, Danmic, USA, 0.40 to 15.00 g). The rats were gently placed under a plastic cage on a metal mesh floor and allowed to move freely. They were acclimated to this environment for approximately 30 min before testing. During the von Frey tests, the rats received a stimulus from filaments on the lateral plantar surface of the operated left hind paw through the mesh floor (Fig. 1A, bottom right). The probe was performed only when the rat’s paw made contact with the floor. Each probe was applied to the foot with sufficient force to bend the filament probe and kept in this position for 6–8 s. The weight of the applied filaments started at 2 g, and the interval between consecutive stimuli was at least 3 min. The mechanical threshold was calculated using the formula described in a previous study [57].

### Dynamic allodynia (brush) test

We performed dynamic allodynia measurement by gently stroking the operated left hind paw from the heel to toe using a paintbrush. The rats were acclimated to the environment in the same manner as in the von Frey test. Sham rats did not respond to the stimuli and were scored as 0. For SNC rats, raising of the foot and returning it to the original position was scored as 1. Lifting of a foot for more than 2 s and placing it back down in another place was scored as 2. If the rat lifted its foot for over 2 s and exhibited a change in body position or another behavioral response, a score of 3 points was given. The test was repeated 3 times at 5-min intervals, and the average of the three scores was calculated [58].

### Pinprick and light touch sensitivity tests

The pretest adaptation was the same as in the von Frey test described above. For the light touch sensitivity test, we lightly stroked the operated left hind paw from the heel to toe using a paintbrush (Fig. 1H, left). If no movement was elicited, the trial was scored as 0; if the rat walked away or lifted the paw briefly, the trial was scored as 1. Stroking was repeated 5 times at 10-s intervals, and the sum of the five tests was used to calculate the final score. In the pinprick test, a pin was used to stroke the paw at 5 points without penetrating the skin (F ig. 1I, left), and the withdrawal responses of the rats in five trials performed at 1-min intervals were counted [59].

### Motor function evaluation

The animal gait analysis system, DigiGait™ (Mouse Specifics Inc., Boston, MA, USA), was used to obtain movement data on passive fast walking after SNC. The rats were compelled to walk/run at a fixed speed and gradient on an enclosed transparent treadmill. Before commencing the experiments, we trained the rats to perform uninterrupted runs (each containing a minimum of 3 step cycles) at a speed not lower than 10 cm/s. For formal trials, each rat was required to complete three uninterrupted runs on the treadmill at intervals of at least 10 min. We selected coordination (swing and duty cycle) and area data for further assessment, and we normalized both sets of data to the healthy hind paw’s data on the side that had not undergone operation (ratio = LH/RH). Swing represents the duration of the swing phase during walking/running. The duty cycle was defined as the time spent in the stance phase divided by the time occupied by a single step. The print area was the maximum projected area calculated by the DigiGait software as the maximum contact area [60].

### Retrograde fluorescein labeling

Two weeks prior to the operation, the left sciatic nerve was exposed as described previously. Using a pulled-glass micropipette, we slowly injected DiI (0.25%, 1 μL, Invitrogen, D275) into the TN and CPN proximal to the prospective crush site and DiO (0.25–0.5%, 0.5 μL Invitrogen, D282) into the SN distal to the prospective crush site, thereby avoiding potential fluorescent dye interactions.

### Acute dissociation of DRG neurons

Rats were subjected to sham or SNC operation followed by fluorescein labeling with DiI (crushed TN/CPN) and DiO (uncrushed SN). At the time points (Additional file 7: Table S1 and Additional file 8: Table S2), sham and SNC rats were deeply anesthetized by intraperitoneal injection of 0.5% pentobarbital sodium, and the L4–L5 DRG from the operated side were harvested. The DRG were immediately treated with collagenase D (3 mg/ml) (Sigma–Aldrich, C9891) at 37 °C for 55 min, followed by treatment with 0.25% trypsin (Gibco, 25,200,056) for 7 min and mechanical trituration using a flame-polished Pasteur pipette. The dissociated neurons were collected, resuspended in DMEM (Gibco, 11,966,025) supplemented with 10% fetal bovine serum (FBS) (HyClone, SH30070.03E), and plated on poly-d-lysine (Sigma–Aldrich, P6407) 35-mm plastic dishes before further study.

### scRNA-seq library preparation and sequencing

A modified Smart-seq2 protocol developed by the Tang laboratory was used for single-cell RNA-seq [23, 24]. Briefly, acutely dissociated DRG neurons were plated on dishes as described previously. After 15 min, the DMEM (Gibco, 11,966,025) was replaced with PBS, and a mouth pipette was then used to place each individual fluorescently labeled single DRG neuron into a volume of lysis buffer under a fluorescence microscope. Harvesting of the single DRG neurons from one rat was completed in 4 h, and the L4–L5 DRG of at least 5 rats were harvested at each time point (Additional file 8: Table S2).

The reverse transcription reaction was performed with a TSO primer added at the 5′ end and with 25 nt oligo (dT) primer anchored with an 8-nt cell-specific barcode and 8-nt unique molecular identifiers (UMIs) added at the 3′ end [61–63]. After reverse transcription, cDNAs were amplified by 15 cycles of PCR, during which the IS primer and the 3′P2 primer were added to the 5′ and 3′ ends of the cDNAs, respectively.

The amplified cDNAs from single cells were pooled, purified using a DNA Clean & concentration-5 kit (Zymo Research, D4014) and AMPure XP beads (Beckman Coulter, A63882), and quantified using a Qubit dsDNA HS Assay Kit (Invitrogen, Q32854). To add biotin tags to the 3′ ends of the amplified cDNAs, biotinylated preindexed primers and IS primer were used to further amplify the PCR product through an additional 4 cycles of PCR. The biotinylated cDNAs were then sheared to approximately 300 bp using Covaris S2, and the 3′ termini of the cDNAs were captured using C1 beads (Dynabeads® Myone™ Streptavidin C1, Invitrogen, 65,001). The RNA-seq library was constructed using a KAPA HyperPrep Kit (KAPA Biosystems, KK8505), and short universal primers and QP2 primers were used in the last amplification. Finally, the genome and transcriptome libraries were subjected to 150-bp paired-end sequencing on an Illumina HiSeq 4000 platform (sequenced by Novogene). Information on the primers used in scRNA-seq is provided in Additional file 9: Table S3.

### Processing of single-cell RNA-seq data

Raw reads were separated based on the specific cell barcode information, and the template switch oligo (TSO) sequence and polyA tail sequence were removed. The clean reads were then aligned to the rat transcriptome (Rnor_6.0.102) using STAR (version 2.7.3b) [64]. Finally, the gene read table was generated using featureCounts, Samtools, and UMI_tools. The sequencing depth of each cell was 0.5 Gbytes.

### QC and unsupervised clustering and visualization

For initial quality control (QC) of 1430 sequenced single cells, we filtered out cells with fewer than 500 genes or 1000 transcripts detected, and limited mitochondrial genes to less than 5%. A total of 1430 cells passed this filter. We then used Seurat (Version: 4.0.3) in R [65] for unsupervised clustering, visualization, and matching of DRG neuronal subtypes. The original reads were normalized to 10,000 transcript reads per cell using the “LogNormalize” processing method.

Subsequently, we applied the FindVariableFeatures() function to screen the top 2000 genes using “variance-stabilizing transformation” as the screening method. To normalize all genes, ScaleData() was applied, and the RunPCA() function was used to preprocess the cluster data. Next, based on the first 40 principal components defined by the JackStraw() function, unsupervised clustering was performed using the FindClusters() function; the cluster resolution was set at 0.5 to determine the number of subgroups and subsets. Finally, the runUMAP() function in Seurat was performed for clustering and visualization based on Uniform Manifold Approximation and Projection (UMAP).

### Matching of DRG neuronal subtypes

Due to the lack of studies on DRG typing in rats, we referred to related studies performed on mouse DRG in 2020 for matching [17, 18]. The DoHeatmap() and DotPlot() functions in Seurat were used to match the DRG neuronal subtype marker genes (genes with relative expression higher than 1.5) identified in those studies; the matching table is shown in the main text.

We combined the 10 cell clusters into 7 subtypes of known DRG cell types, including PEP1 (*Tac1*^+^/*Gal*^+^), PEP2 (*Gal*^+^ with low *Tac1*^+^ expression), PEP3 (*Tac1*^+^/*Gpx3*^+^ with low *Gal*^+^ expression), NP (*Scg3*^+^ with low *Sst*^+^ expression), NF (*Nefh*^+^), SST (*Sst*^+^), and c_pLTMR2 (*Zfp521*^+^ with low *Th*^+^ expression). A certain amount of overlap and ambiguity in the clustering could not be avoided due to the total number of cells and the lack of previous identification of effective characteristic DRG neuronal subtype matching genes in rats. Given our random sampling of DRG single neurons, the proportions of different subgroups that we observed may partially reflect changes in the proportions of DRG neuronal subtypes that occur after injury; according to the GraphPad plot analysis, the proportion of PEP neurons changed greatly with time.

### Gene Ontology (GO) and Kyoto Encyclopedia of Genes and Genomes (KEGG) analysis

FindAllMarkers() in Seurat was applied to identify cell type-specific genes, and Metascape [25] was used to analyze gene function in the three PEP DRG neuronal groups.

### Experimental design scRNA sequence processing (EDSSP)

All cell-derived data in PEP1 were preprocessed using the vst() function of Deseq2 in R [66]. Based on the experimental design, all the DRG neurons in PEP1 were used to create a dataset, and WGCNA in R [26, 27, 67] was then applied to identify gene co-expression modules in the transcriptomes of differentially expressed genes. In general, the WGCNA self-contained one-step method was used to generate co-expression gene modules (power = 3), dendrograms, and correlation matrix heatmaps. Correlation analysis was performed on the dataset created in this experiment using the script that expresses transcript set annotations [67]. After exporting the gene co-expression module, GO and KEGG analyses were performed as described above. Finally, key modules were further intersected with cell type-specific genes to identify vital genes with potential biological properties.

### qPCR and single-cell qPCR

Total RNA was extracted from the L4–L5 DRG of rats using an RNA isolation kit (Aidlab, RN28) and then reverse-transcribed using RT Master Mix (AbmGood, G490). qPCR was then performed using SYBR® Green Realtime PCR Master Mix (Toyobo, QPK-201) in a 7500 Real-Time PCR System (Applied Biosystems). Relative quantification of target gene expression was performed after normalization to the mRNA level of Gapdh by the comparative cycle threshold method. The data are presented as the mean ± SEM.

For single-cell qPCR of DRG neurons, the amplified cDNAs remaining after the first purification were kept for validation experiments. The amplified cDNAs were first purified using AMPure XP beads and amplified by 9 cycles of PCR. Before qPCR, the newly amplified cDNAs were purified again using AMPure XP beads to remove the residues from PCR. qPCR and data analysis were performed in the same way as described above. For information on the primers used in qPCR, see Additional file 10: Table S4.

### Immunofluorescence staining

While under the anesthesia of 0.5% pentobarbital sodium, rats were perfused with warm saline followed by cold 4% PFA. After perfusion, the sciatic nerves were isolated and immersed in 4% PFA at 4 °C for 4–6 h, and the medial plantar glabrous footpad skin was immersion-fixed in Zamboni’s fixative at 4 °C overnight. All the samples were dehydrated in 30% (w/v) sucrose in 0.1 M PBS for cryoprotection and preserved at 4 °C before cryosectioning. Frozen sections of sciatic nerves were cut with a cryostat to a thickness of 10 μm and then mounted directly onto gelatin-coated slides. Frozen sections of glabrous footpad skin were cut at 20 μm in a cryostat and immediately mounted on gelatin-coated slides. The sections were then incubated with primary antibodies (anti-GAP43, Abcam, Ab16053, 1:500; anti-PGP9.5, AbD Serotec, 7863–1004, 1:100; anti-NF200, Sigma-Aldrich, N0142, 1:200) at 4 °C for 24 h, followed by incubation with the appropriate HRP-conjugated fluorescent antibody (Alexa Fluor 488-conjugated goat anti-rabbit, Invitrogen, A-11034,1:500; Alexa Fluor 488-conjugated donkey anti-mouse, Invitrogen, A-21202, 1:500; Alexa Fluor 594-conjugated donkey anti-mouse, Invitrogen, A-21203, 1:500) and Hoechst 33,342 or DAPI staining. The images were photographed by confocal laser scanning microscopy (Leica TCS SP8 STED). Images were acquired using an inverted Leica true confocal scanner (TCS) SP8 microscope.

To quantify the regenerative nerve fibers in the glabrous footpad skin, based on the structure of the skin, three zones were defined (Additional file 1: Fig. S1). Zone 1 is defined as the dermis layer in which the subepidermal nerve plexus length (SNPL) was determined and divided by the length of the epidermis. Zone 2 and zone 3 are defined based on the boundary between stratum granulosum (SG) and stratum spinosum (SS) sub-layers in the epidermis. The density of the epidermal nerve fibers was analyzed by counting the numbers of nerve fibers per 0.3 mm of epidermis. Nerve fibers branching in zone 2 were counted separately, while nerve fibers branching in zone 3 were counted as one.

### Simultaneous RNAscope and immunofluorescence assay

To visualize and quantify *Adcyap1* expression, an RNAscope probe targeting *Adcyap1* was designed and synthesized by Advanced Cell Diagnostics. The RNAscope and immunofluorescence (IF) assays were performed using RNAscope® Multiplex Fluorescent Reagent Kit v2. For each experiment, *POLR2A* served as the positive control, while *dapB* was the negative control.

Briefly, after fixing in 4% PFA for 24 h, fresh rat DRG Sects. (10-μm thin) were prepared and then pretreated with hydrogen peroxide solution, target retrieval solution, and stained with primary antibodies overnight at 4 °C. The sections were further treated with protease plus and finally hybridized with the RNA probe of the target gene for 2 h at 45 °C in a hybrid furnace, followed by a series of signal amplifications. After RNAscope, the sections were stained with antibody (Invitrogen, PA5-17,646) for 30 min at room temperature. The nuclei were counterstained with DAPI for 10 min at room temperature. Images were obtained with an Olympus FV3000 confocal microscope.

The signal dots were visually counted using QuPath (version 0.4.3) [68] following the RNAscope manual (as outlined at https://acdbio.com/2022-jul-26-visualization-and-analysis-rnascope%E2%84%A2-results-using-qupath), and the single dot represents a single mRNA.

### Intrathecal catheterization and injection

To administer drugs intrathecally, a PE-10 polyethylene catheter was inserted into the intrathecal space above the lumbar enlargement of the spinal cord [69]. The rats were anesthetized with isoflurane during the operation and allowed to recover for 4 days before the SNC operation was performed. Rats with motor impairment caused by intrathecal catheterization were excluded from the study, and the basal nociceptive responses of animals were measured before group allocation, which was performed randomly.

For siRNA administration, rats were given 4 μg *Adcyap1* siRNA or control siRNA every other day from 2 days post-injury (dpi); the siRNA was mixed with in vivo jetPEI® as described in the in vivo jetPEI® Protocol.

The animals’ nociceptive responses were assessed at 1 and 3 dpi, and L4–L5 DRG from rats were harvested to check the efficiency of knockdown by qPCR. The sequences of the control siRNAs [70] were sense 5′-UUCUCCGAACGUGUCACGUTT-3′ and antisense 5′-ACGUGACACGUUCGGAGAATT-3′. The sequences of the *Adcyap1* siRNAs were sense 5′-GGAUAAUAAUGCAUAACAGTT-3′ and antisense 5′-CUGUUAUGCAUUAUUAUCCTT-3′. The siRNAs were modified with 2′ OME and 5′-Chol and synthesized by GenePharma.

For peptide delivery, after 4 days of recovery from the intrathecal catheterization, rats received intrathecal injections of peptides. For administration of PACAP6-38, scramble or PACAP6-38 peptides were intrathecally injected at various dosages: 4 μg, 13 μg, and 40 μg. The animals’ nociceptive responses were assessed at 1 dpi. The sequence of PACAP6-38 was FTDSYSRYRKQMAVKKYLAAVLGKRYKQRVKNK. The sequence of the scramble peptide was ASSKVKRRYVYYTKKKQKVMAGYKNRLDRQFAL. For the administration of PACAP38, 15 μg scramble or PACAP38 peptides were intrathecally injected into SNC rats, and the animals’ nociceptive responses were assessed at 7, 14, and 21 dpi. The sequence of PACAP38 was HSDGIFTDSYSRYRKQMAVKKYLAAVLGKRYKQRVKNK. The sequence of the scramble peptide was YFYKQSKIDKADYKVVKKGRRMAKTLYLRHQGNSVSRA. The peptides were synthesized and purified by GL Biochem Ltd. (Shanghai, China).

### DRG neuron culture

DRG neurons from naïve rats and from rats that had been intrathecally injected with siRNA were acutely dissociated and plated on dishes as described previously. The dissociated neurons were collected, resuspended in DMEM (Gibco, 11,966,025) supplemented with 10% fetal bovine serum (FBS) (HyClone, SH30070.03E), and plated on 35-mm plastic dishes coated with poly-d-lysine (Sigma–Aldrich, P6407). Three hours after plating, the DMEM (Gibco, 11,966,025) was removed, and neurobasal medium (Gibco, 21,103,049) containing 2% B-27 (Gibco, 17,504,044), 2 mM GlutaMAX™-I (Gibco, 35,050,061), and 5 μM cytarabine (Sigma–Aldrich, C6645) was added; the cells were then cultured in the latter medium until the end of the experiments. DRG neurons from rats that had been intrathecally injected with Adcyap1 or scramble siRNA were cultured for 2 days before morphological analysis. Cultured DRG neurons from naïve rats were incubated with 10 nmol PACAP6-38 or scramble peptide in a neurobasal medium (Gibco, 21,103,049) for 3 h after plating and then cultured for 3 days before further study, and for PACAP38, cultured DRG neurons from naïve rats were incubated with 3.33 nmol PACAP38 or scramble peptide in neurobasal medium (Gibco, 21,103,049) for 3 h after plating and then cultured for 2 days before further study.

### Estimation of neurite lengths

After several days of culture, the medium in which clustered DRG neurons were maintained was replaced with 4% PFA, and the cultures were washed with PBS. The DRG neurons were then incubated with primary antibodies (rabbit anti-beta3-tubulin, Cell Signaling Technology, D71G9, 1:500) at 4 °C overnight, followed by incubation with an HRP-conjugated secondary antibody (Alexa Fluor 488-conjugated goat anti-rabbit, Thermo Fisher Scientific, A-11034, 1:500). The cultures were then viewed and photographed using an inverted fluorescence microscope (Leica), and the lengths of the neurites produced by the DRG neurons were manually measured using the ImageJ 1.53c software (Wayne Rasband, National Institutes of Health).

### Statistical analyses

All data are presented as mean ± SEM. Statistical analyses were performed using Prism 8.0 software (GraphPad Software, La Jolla, CA, USA). Comparisons between the groups were performed using either Student’s unpaired *t* test with two-way ANOVA followed by Sidak’s multiple-comparisons test or one-way ANOVA with Sidak’s multiple-comparisons test. Statistical significance was set at *P* < 0.05.

### Supplementary Information

**Additional file 1: Fig. S1.** Crushed sensory axons regrowth of SNC rats.

**Additional file 2: Fig. S2.** Sc-RNA sequencing quality control shows that the scRNA-seq data obtained in this study are suitable for deeper analysis.

**Additional file 3: Fig. S3.** PEP1 and PEP3 highly-expressed genes in all-clusters gene expression and validation of PEP1 subtype’s marker genes.

**Additional file 4: Fig. S4.** The metascape results of PEPs.

**Additional file 5: Fig. S5.** Results of WGCNA and metascape results of 3 module genes.

**Additional file 6: Fig. S6.** PACAP38 promotes axonal outgrowth of DRG neurons in vitro and attenuates mechanical allodynia after SNC.

**Additional file 7: Table S1.** The statistics of 10 observed rat DRG neuron clusters with time and surgical information.

**Additional file 8: Table S2.** Information on DRG samples.

**Additional file 9: Table S3.** Information on qPCR primer sequences.

**Additional file 10: Table S4.** Information on the overlap between 4 gene modules and PEP cluster marker genes.

## Data Availability

Raw and processed data are also deposited within the Gene Expression Omnibus (GEO) repository (https://www.ncbi.nlm.nih.gov/geo) with an accession number (GSE198608). All software is freely or commercially available and is listed in the “ Methods” section description. All data generated or analyzed during this study are included in this published article and its supplementary information files.

## Abbreviations

AUC: Area under the curve
c_pLTMR2: Putative low-threshold c-mechanoreceptors
CNI: Central nerve injury
CPN: Common peroneal nerve
dpi: Days post-injury
DRG: Dorsal root ganglion
DRGs: Dorsal root ganglion neurons
EDSSP: Experimental design scRNA sequence processing
FBS: Fetal bovine serum
Gal: Galanin
GEO: Gene Expression Omnibus
GO: Gene Ontology
hiPSCd: Human induced pluripotent stem cell-derived
IVC: In ventilated cages
KEGG: Kyoto Encyclopedia of Genes and Genomes
LH/RH: Left hind/right hind
NFs: Nefh + A-fiber low-threshold mechanoreceptors
NPs: Nonpeptidergic nociceptors
PAC1R: Pituitary adenylate cyclase 1 receptor
PACAP: Pituitary adenylate cyclase-activating polypeptide
PEPs: Peptidergic nociceptors
PNI: Peripheral nerve injury
QC: Quality control
Scg3: Secretogranin III
scRNA-seq: Single-cell RNA sequencing
SD: Sprague-Dawley
SG: Stratum granulosum
SGs: Secretory granules
SN: Sural nerve
SNC: Spared nerve crush
SNI: Spared nerve injury
SNPL: Subepidermal nerve plexus length
SPF: Specific pathogen-free
SS: Stratum spinosum
SSTs: Somatostatin + pruriceptors
TCS: True confocal scanner
TN: Tibial nerve
TSO: Template switch oligo
UMAP: Uniform manifold approximation and projection
UMIs: Unique molecular identifiers
wpi: Weeks post-injury
