# Supplementary material for: Unveiling adcyap1 as a protective factor linking pain and nerve regeneration through single-cell RNA sequencing of rat dorsal root ganglion neurons
Source: BMC Biol. 2023 Oct 25;21:235. doi: 10.1186/s12915-023-01742-8 (PMC10601282; doi:10.1186/s12915-023-01742-8)
Supplement: Supplementary file 1 — Additional file 1: Fig. S1. Crushed sensory axons regrowth of SNC rats. [file 12915_2023_1742_MOESM1_ESM.pdf]

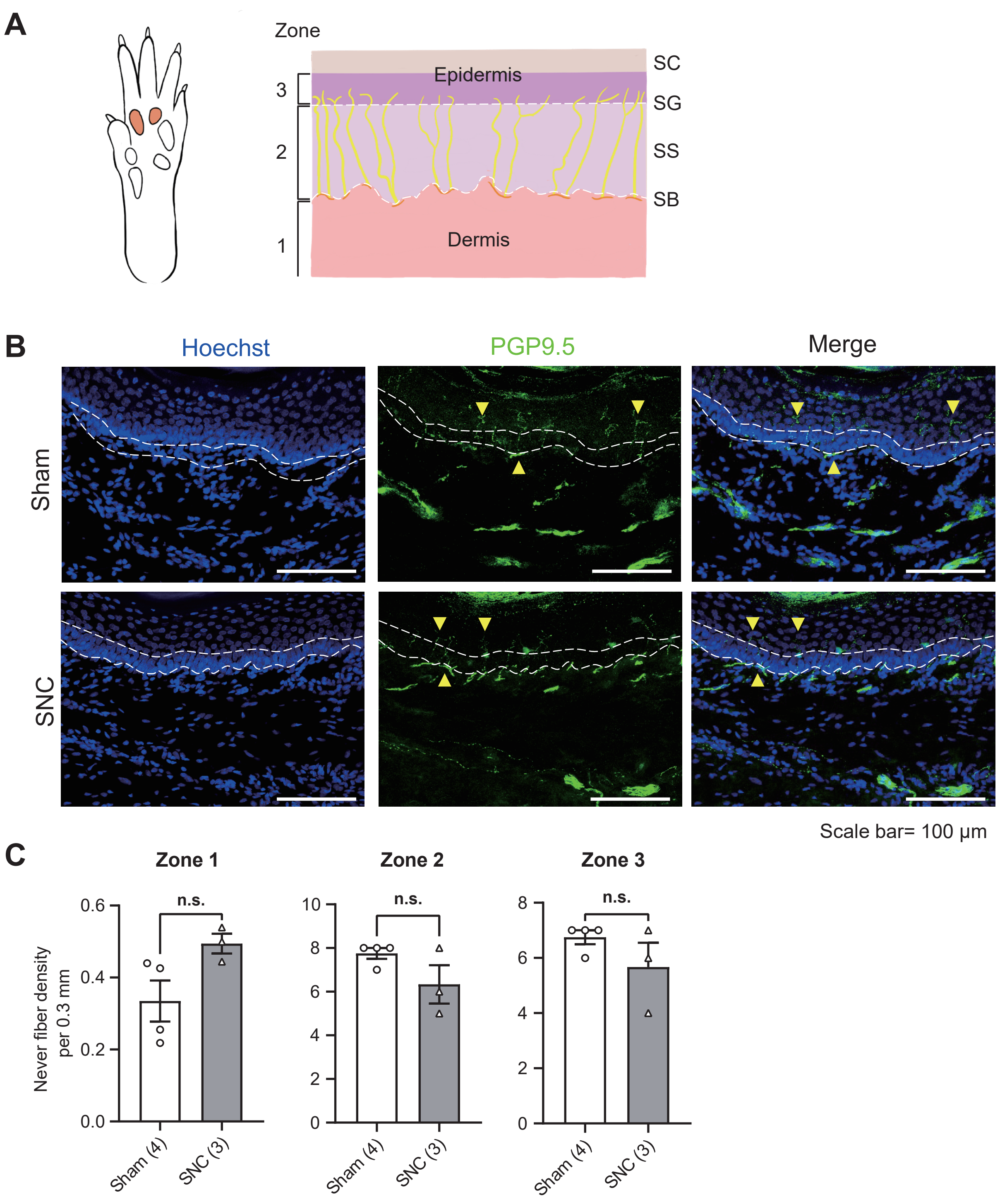

Supplementary Fig. 1 Crushed sensory axons regrowth of SNC rats. (**A**) Schematic diagram of the glabrous footpad regions in rat skin samples (left) cross sections of hindpaw glabrous skin tissue images (right). (**B**) Representative images of nerve fibers in glabrous footpad skin of the ipsilateral hindpaw of animals in the sham and SNC groups in 28 days post injury (dpi), immunostained for PGP9.5 with hoechst staining (yellow arrows: nerve fibers, dotted lines: borders between three defined zones of skin). SC: stratum corneum; SG: stratum granulosum; SS: stratum spinosum; SB: stratum basale. Scale bar, 100 μm. (**C**) Quantifications of the number of nerve fibers in a 0.3 mm segment of epidermal areas in sham (n= 4) and SNC (n= 3) groups. Data are represented as mean ± SEM. Unpaired t test, n.s., no significance difference.
