# Supplementary material for: Unveiling adcyap1 as a protective factor linking pain and nerve regeneration through single-cell RNA sequencing of rat dorsal root ganglion neurons
Source: BMC Biol. 2023 Oct 25;21:235. doi: 10.1186/s12915-023-01742-8 (PMC10601282; doi:10.1186/s12915-023-01742-8)
Supplement: Supplementary file 2 — Additional file 2: Fig. S2. Sc-RNA sequencing quality control shows that the scRNA-seq data obtained in this study are suitable for deeper analysis. [file 12915_2023_1742_MOESM2_ESM.pdf]

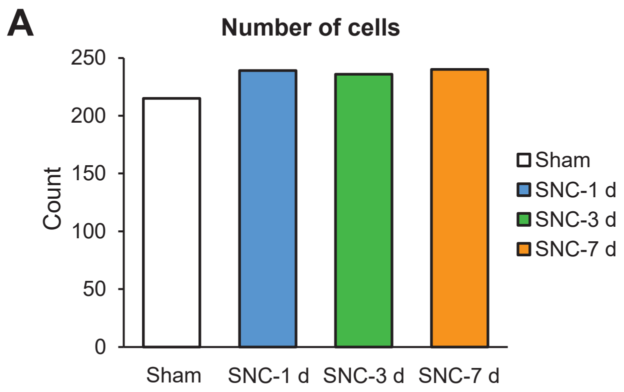


| | Sham | SNC-1 d | SNC-3 d | SNC-7 d |
|---|---|---|---|---|
| **Red** | 108 | 111 | 116 | 120 |
| **Green** | 107 | 128 | 120 | 120 |
| **Total** | 215 | 239 | 236 | 240 |

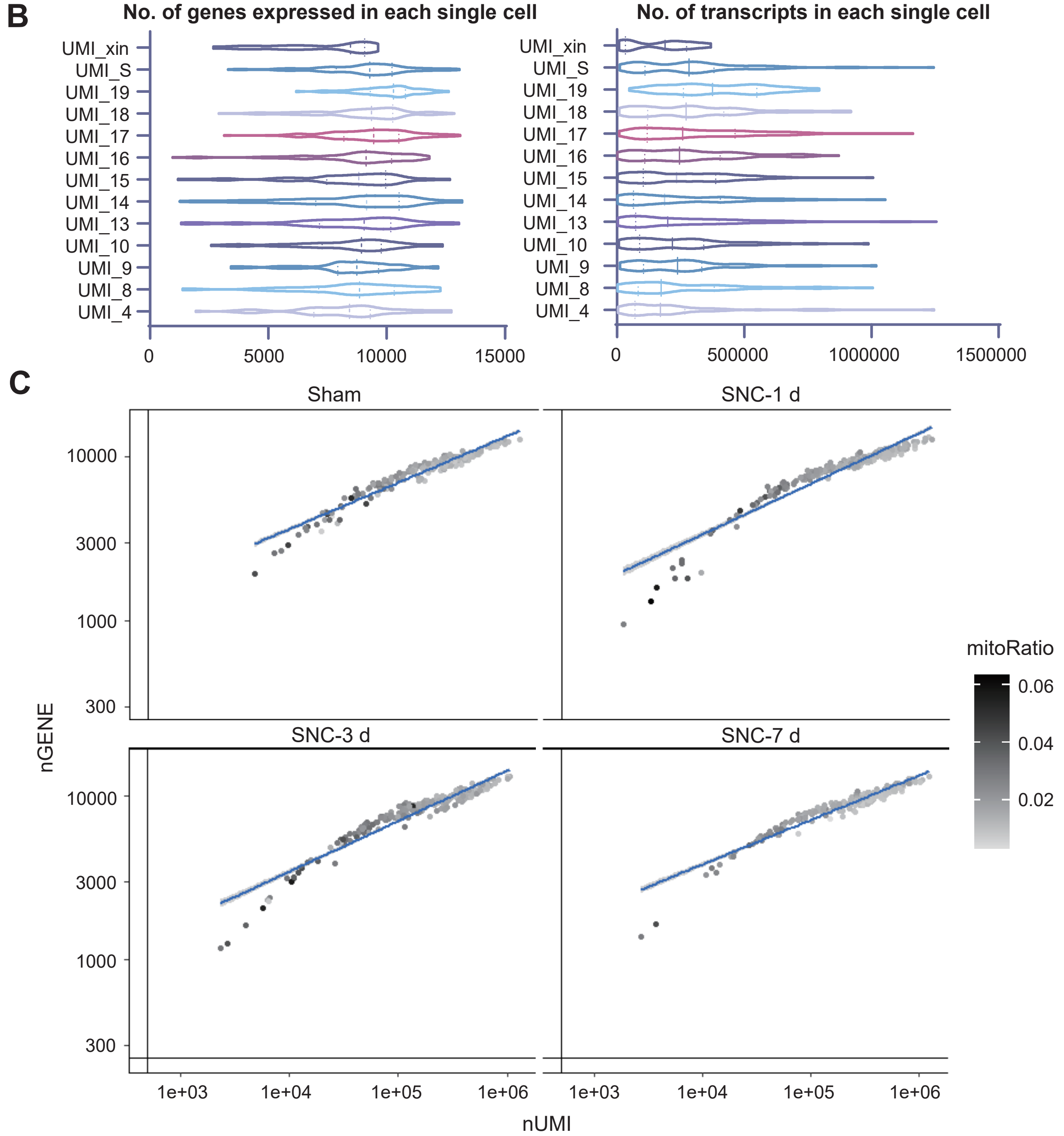


**Supplementary Fig. 2 Sc-RNA sequencing quality control shows that the scRNA-seq data obtained in this study are suitable for deeper analysis.** (**A**) The statistics of qualified cells and the sheet of experimental design. (**B** and **C**) The QC results of scRNA-seq data across batches and time-design.
