# Supplementary material for: Unveiling adcyap1 as a protective factor linking pain and nerve regeneration through single-cell RNA sequencing of rat dorsal root ganglion neurons
Source: BMC Biol. 2023 Oct 25;21:235. doi: 10.1186/s12915-023-01742-8 (PMC10601282; doi:10.1186/s12915-023-01742-8)
Supplement: Supplementary file 3 — Additional file 3: Fig. S3. PEP1 and PEP3 highly-expressed genes in all-clusters gene expression and validation of PEP1 subtype’s marker genes. [file 12915_2023_1742_MOESM3_ESM.pdf]

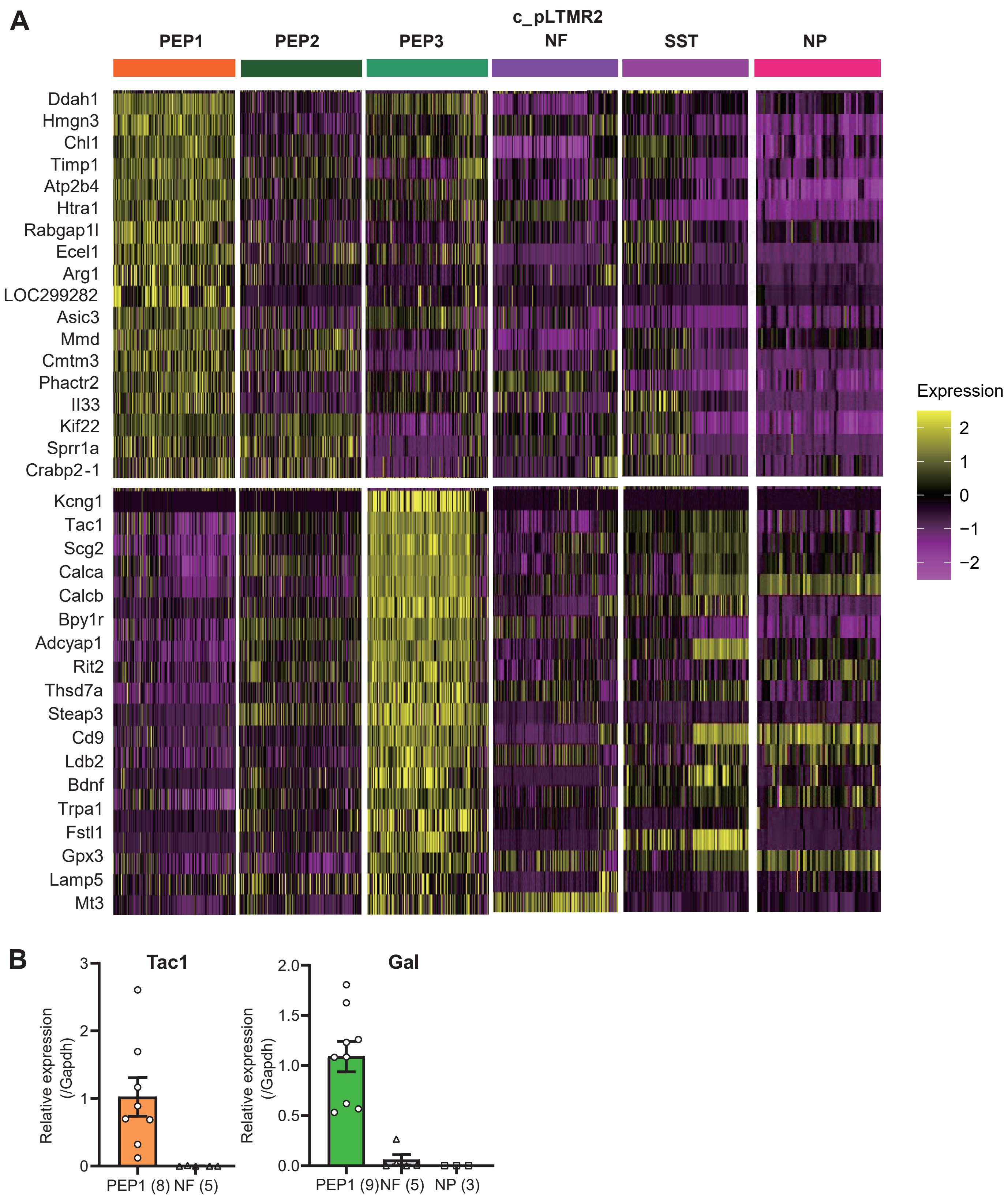

Supplementary Fig. 3 PEP1 and PEP3 highly-expressed genes in all-clusters gene expression and validation of PEP1 subtype's marker genes. (**A**) The heatmap of all-clusters gene expression in PEP1 and PEP3 highly-expressed genes. (**B**) Single cell qPCR results of PEP1 subtype's marker genes: Tac1 (left; n= 8 for PEP1 DRGs and n= 5 for NF DRGs) and Gal (right; n= 9 for PEP1 DRGs, n= 5 for NF DRGs, and n= 3 for NP DRGs), Data are represented as mean ± SEM.
