# Supplementary material for: Unveiling adcyap1 as a protective factor linking pain and nerve regeneration through single-cell RNA sequencing of rat dorsal root ganglion neurons
Source: BMC Biol. 2023 Oct 25;21:235. doi: 10.1186/s12915-023-01742-8 (PMC10601282; doi:10.1186/s12915-023-01742-8)
Supplement: Supplementary file 4 — Additional file 4: Fig. S4. The metascape results of PEPs. [file 12915_2023_1742_MOESM4_ESM.pdf]

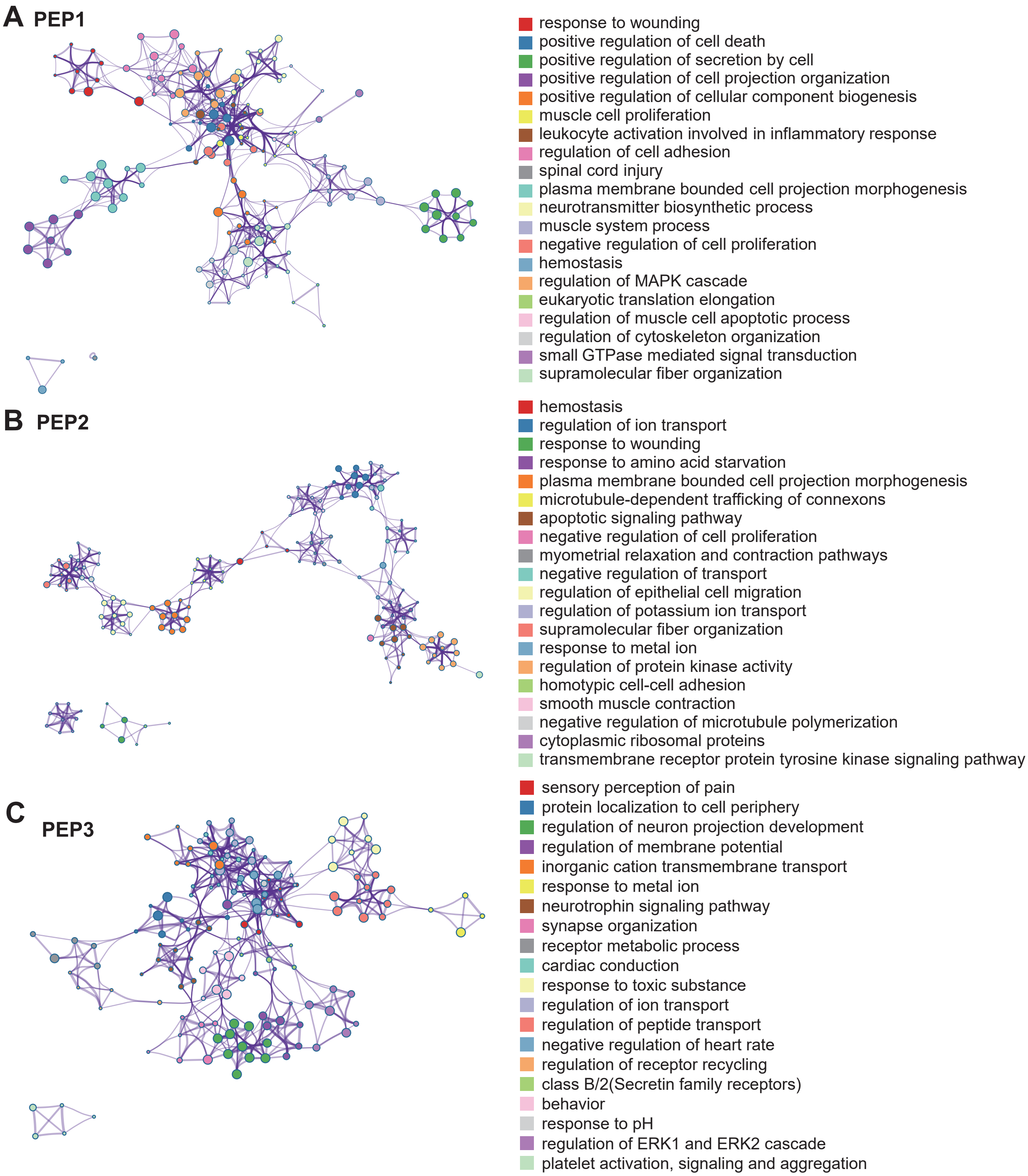

Supplementary Fig. 4 The metascape results of PEPs. (**A**) The metascape analysis of highly-expressed genes in PEP1 reveals that PEP1 is highly-related with injury and repair processing. (**B**) Metascape results of PEP2 is highly-related with complex cell processing. (**C**) Metascape results of PEP3 reveals that PEP3 is highly-related with pain.
