# Supplementary material for: Unveiling adcyap1 as a protective factor linking pain and nerve regeneration through single-cell RNA sequencing of rat dorsal root ganglion neurons
Source: BMC Biol. 2023 Oct 25;21:235. doi: 10.1186/s12915-023-01742-8 (PMC10601282; doi:10.1186/s12915-023-01742-8)
Supplement: Supplementary file 5 — Additional file 5: Fig. S5. Results of WGCNA and metascape results of 3 module genes. [file 12915_2023_1742_MOESM5_ESM.pdf]

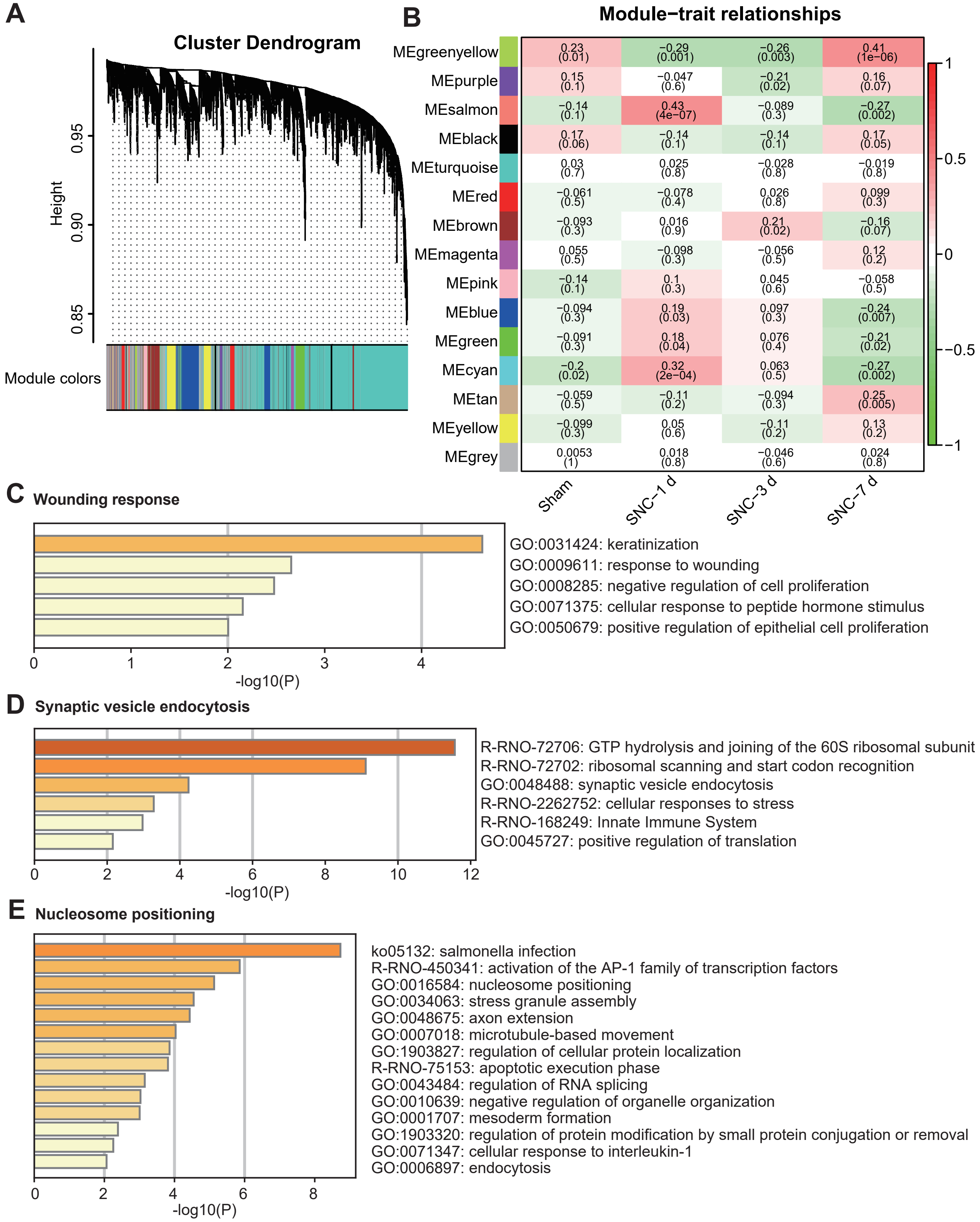

Supplementary Fig. 5 Results of WGCNA and metascape results of 3 module genes. (**A**) Cluster dendrogram for all the crushed PEP1 DRG neurons. (**B**) Module-trait relationships reveals that there are 4 modules of genes changed by time-design. (**C** to **E**) The metascape results of Wounding response, Synaptic vesicle endocytosis and Nucleosome positioning's module genes.
