# Supplementary material for: Unveiling adcyap1 as a protective factor linking pain and nerve regeneration through single-cell RNA sequencing of rat dorsal root ganglion neurons
Source: BMC Biol. 2023 Oct 25;21:235. doi: 10.1186/s12915-023-01742-8 (PMC10601282; doi:10.1186/s12915-023-01742-8)
Supplement: Supplementary file 6 — Additional file 6: Fig. S6. PACAP38 promotes axonal outgrowth of DRG neurons in vitro and attenuates mechanical allodynia after SNC. [file 12915_2023_1742_MOESM6_ESM.pdf]

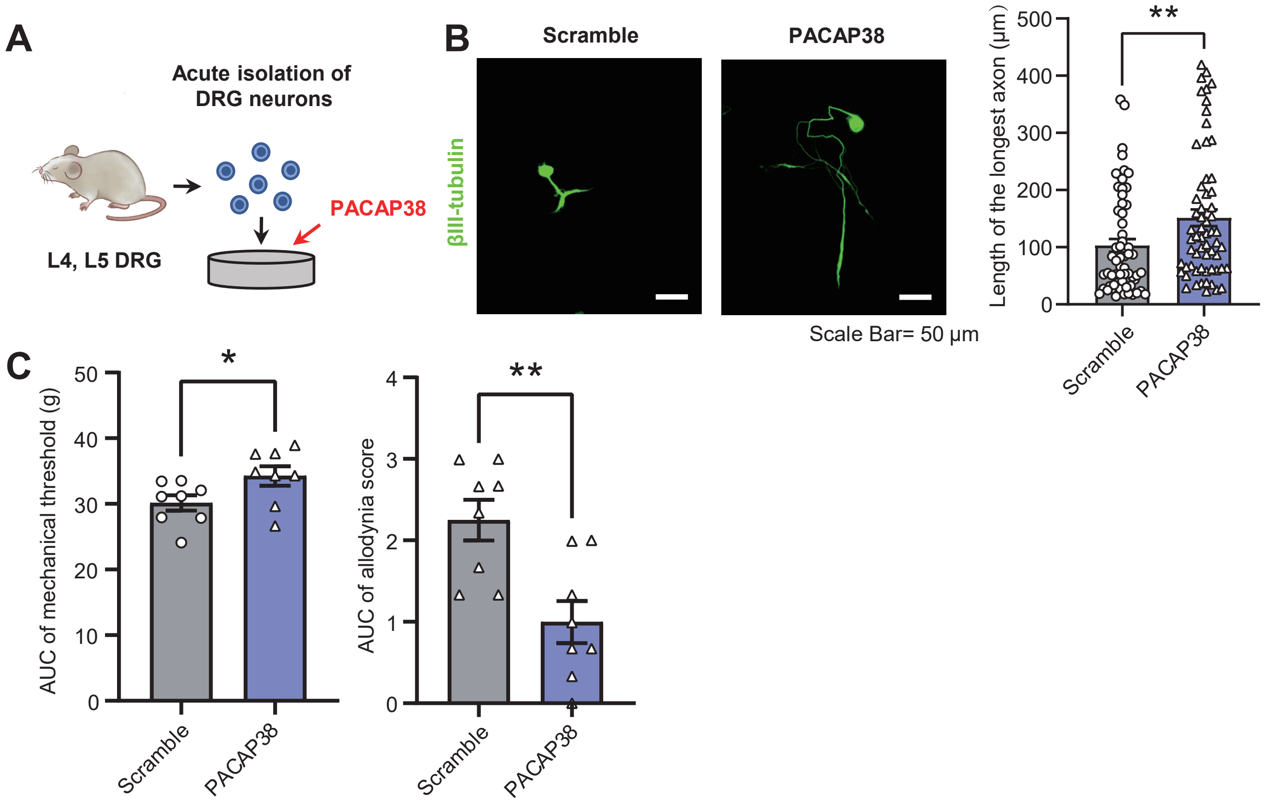

Supplementary Fig. 6 PACAP38 promotes axonal outgrowth of DRG neurons in vitro and attenuates mechanical allodynia after SNC. (**A**) Schematic diagram of the protocol for the investigation of axonal outgrowth of DRG neurons clustered with Pacap38. (**B**) Representative images and quantification of axonal outgrowth in acute-dissociated DRG neurons from naïve rats cultured with scramble (n= 3) or PACAP38 (n= 3) for 2 days, immunostained for βIII-tubulin. Scale bar, 50 μm. (**C**) The area under curve (AUC) mechanical threshold (left) and allodynia score (right) analysis (1-21 day) of the ipsilateral hind paw after intrathecal administration of scramble (n= 8) or PACAP38 (n= 8) in the acute phase of SNC. Data are represented as mean ± SEM, unpaired t test in (B) and (C), $*P < 0.05$, $**P < 0.01$.
