# Supplementary material for: Unveiling adcyap1 as a protective factor linking pain and nerve regeneration through single-cell RNA sequencing of rat dorsal root ganglion neurons
Source: BMC Biol. 2023 Oct 25;21:235. doi: 10.1186/s12915-023-01742-8 (PMC10601282; doi:10.1186/s12915-023-01742-8)
Supplement: Supplementary file 8 — Additional file 8: Table S2. Information on DRG samples. [file 12915_2023_1742_MOESM8_ESM.pdf]

Additional file 8. Table S2. Information on DRG samples

DRG sample	Cell number (R/G)	Time point	RT date	Sequencing date
Xin	12/12	Sham	2019.11.15	2019.12.02
S	48/48	SNC 7 d	2019.11.26	2019.12.02
4	24/24	Sham	2019.12.20	2021.01.18
8	24/24	SNC 7 d	2019.12.20	2021.01.18
9	24/24	SNC 7 d	2019.12.21	2021.01.18
10	24/24	Sham	2019.12.21	2021.01.18
13	48/48	SNC 1 d	2020.11.27	2021.01.18
14	48/48	SNC 3 d	2020.12.04	2021.01.18
15	46/50	SNC 3 d	2020.12.08	2021.01.18
16	45/51	SNC 1 d	2020.12.09	2021.01.18
17-1	12/12	SNC 1 d	2020.12.25	2021.01.18
17-2	8/16	SNC 1 d	2020.12.25	2021.01.18
17-3	22/26	SNC 3 d	2020.12.25	2021.01.18
18	48/48	Sham	2020.12.29	2021.01.18
19-1	4/20	SNC 7 d	2020.12.25	2021.01.18
19-2	20/4	SNC 7 d	2020.12.25	2021.01.18

* RT: reverse transcription
