# Supplementary material for: Unveiling adcyap1 as a protective factor linking pain and nerve regeneration through single-cell RNA sequencing of rat dorsal root ganglion neurons
Source: BMC Biol. 2023 Oct 25;21:235. doi: 10.1186/s12915-023-01742-8 (PMC10601282; doi:10.1186/s12915-023-01742-8)
Supplement: Supplementary file 9 — Additional file 9: Table S3. Information on qPCR primer sequences. [file 12915_2023_1742_MOESM9_ESM.pdf]

Additional file 9. Table S3. Information on qPCR primer sequences

Primer name	Direction	Sequence
Gapdh	Sense	AAGGGCTCATGACCACAGTC
	Antisense	GGATGCAGGGATGATGTTCT
Adcyap1	Sense	GAGAGATGTCGCCCACGAAA
	Antisense	GCGAGGTTCTCGCCCAT
CD9	Sense	GGCTATACCCACAAGGACGA
	Antisense	GCTATGCCACAGCAGTTCAA
Tac1	Sense	GCTCTTTTGCCTATTAGTCC
	Antisense	GCCCTTTGAGCATCTTCTTCA
Gal	Sense	CACATGCCATTGACAACCAC
	Antisense	AACTCCATTATAGTGCGGACG
