# Supplementary material for: Unveiling adcyap1 as a protective factor linking pain and nerve regeneration through single-cell RNA sequencing of rat dorsal root ganglion neurons
Source: BMC Biol. 2023 Oct 25;21:235. doi: 10.1186/s12915-023-01742-8 (PMC10601282; doi:10.1186/s12915-023-01742-8)
Supplement: Supplementary file 10 — Additional file 10: Table S4. Information on the overlap between 4 gene modules and PEP cluster marker genes. [file 12915_2023_1742_MOESM10_ESM.pdf]

Additional file 10. Table S4. Information on the overlap between 4 gene modules and PEP cluster marker genes

Gene name	Gene id	Overlap	Focus time
ENSRNOG00000001657	Cldnd1	C-1	SNC 1 d
ENSRNOG00000004687	Thbd	C-1	SNC 1 d
ENSRNOG00000004925	Ppp1r12a	C-1	SNC 1 d
ENSRNOG00000008981	Pdcd6ip	C-1	SNC 1 d
ENSRNOG00000010161	Myo10	C-1	SNC 1 d
ENSRNOG00000013304	Arg1	C-1	SNC 1 d
ENSRNOG00000018507	Gfpt1	C-1	SNC 1 d
ENSRNOG00000019357	Tax1 bp3	C-1	SNC 1 d
ENSRNOG00000049976	Gzmb	C-1	SNC 1 d
ENSRNOG00000000521	Cdkn1a	C-1-2	SNC 1 d
ENSRNOG00000002436	Mmd	C-1-2	SNC 1 d
ENSRNOG00000003256	Ccng1	C-1-2	SNC 1 d
ENSRNOG00000016166	Pdlim1	C-1-2	SNC 1 d
ENSRNOG00000016630	Tln1	C-1-2	SNC 1 d
ENSRNOG00000020136	Tgm1	C-1-2	SNC 1 d
ENSRNOG00000024028	Sprr1a	C-1-2	SNC 1 d
ENSRNOG00000002520	Litaf	C-2	SNC 1 d
ENSRNOG00000005708	Mmp16	C-2	SNC 1 d
ENSRNOG00000012422	Tnik	C-2	SNC 1 d
ENSRNOG00000017609	Cnga4	C-2	SNC 1 d
ENSRNOG00000022710	Prrg4	C-2	SNC 1 d
ENSRNOG00000051952	Tes	C-2	SNC 1 d
ENSRNOG00000001628	Pcp4	S-1	SNC 1 d
ENSRNOG00000019704	Resp18	S-1	SNC 1 d
ENSRNOG00000005700	Nsg1	S-1-2	SNC 1 d
ENSRNOG00000008656	Snca	S-1-2	SNC 1 d
ENSRNOG00000012630	Rhoc	S-1-2	SNC 1 d

ENSRNOG00000013496	Crisp3	S-1-2	SNC 1 d
ENSRNOG00000007374	Tac1	S-1-2-3	SNC 1 d
ENSRNOG00000019556	Cd9	S-1-3	SNC 1 d
ENSRNOG00000049882	Adcyap1	S-1-3	SNC 1 d
ENSRNOG00000001338	Hpd	S-2	SNC 1 d
ENSRNOG00000005046	Tspan13	S-2	SNC 1 d
ENSRNOG00000008301	Tagln2	S-2	SNC 1 d
ENSRNOG00000010549	Tspo	S-2	SNC 1 d
ENSRNOG00000023077	Cpne9	S-2	SNC 1 d
ENSRNOG00000000701	Iscu	S-3	SNC 1 d
ENSRNOG00000002746	Fstl1	S-3	SNC 1 d
ENSRNOG00000003205	Ldb2	S-3	SNC 1 d
ENSRNOG00000003927	Cd55	S-3	SNC 1 d
ENSRNOG00000004411	Tspan8	S-3	SNC 1 d
ENSRNOG00000005457	Lamp5	S-3	SNC 1 d
ENSRNOG00000007256	Necab1	S-3	SNC 1 d
ENSRNOG00000007354	Trpa1	S-3	SNC 1 d
ENSRNOG00000008001	Rab3b	S-3	SNC 1 d
ENSRNOG00000011074	Calcb	S-3	SNC 1 d
ENSRNOG00000011130	Calca	S-3	SNC 1 d
ENSRNOG00000011501	Atp1b3	S-3	SNC 1 d
ENSRNOG00000013572	Lxn	S-3	SNC 1 d
ENSRNOG00000014149	Npy1r	S-3	SNC 1 d
ENSRNOG00000015055	Scg2	S-3	SNC 1 d
ENSRNOG00000017568	Rit2	S-3	SNC 1 d
ENSRNOG00000018958	Mt3	S-3	SNC 1 d
ENSRNOG00000019486	Trpv1	S-3	SNC 1 d
ENSRNOG00000021866	Bola3	S-3	SNC 1 d
ENSRNOG00000032473	Scn10a	S-3	SNC 1 d
ENSRNOG00000037853	Rarres1	S-3	SNC 1 d

ENSRNOG00000042753	Fgf13	S-3	SNC 1 d
ENSRNOG00000052564	Gpx3	S-3	SNC 1 d
ENSRNOG00000020684	Vat1	G-1	SNC 7 d
ENSRNOG00000005438	Pcsk2	G-3	SNC 7 d
ENSRNOG00000025881	Rbms3	G-3	SNC 7 d
ENSRNOG00000009088	Txnrd1	T-1	SNC 7 d
ENSRNOG00000011696	Lifr	T-1	SNC 7 d
ENSRNOG00000016047	Macf1	T-1-2	SNC 7 d
ENSRNOG00000026293	Jun	T-1-2	SNC 7 d
ENSRNOG00000014791	Peg3	T-1-3	SNC 7 d
ENSRNOG00000015496	Tpm4	T-2	SNC 7 d
ENSRNOG00000047459	H1f4	T-2	SNC 7 d
ENSRNOG00000057569	Ahnak	T-2	SNC 7 d
ENSRNOG00000002079	Mapk10	T-3	SNC 7 d
ENSRNOG00000024852	Tmx4	T-3	SNC 7 d

* 1,2,3 - PEP1, PEP2, PEP3

** C - Cyan module, S - Salmon module, G - Greenyellow module, T - Tan module
